# Clinicopathologic characteristics of *EGFR*, *KRAS*, and *ALK* alterations in 6,595 lung cancers

**DOI:** 10.18632/oncotarget.8074

**Published:** 2016-03-14

**Authors:** Boram Lee, Taebum Lee, Se-Hoon Lee, Yoon-La Choi, Joungho Han

**Affiliations:** ^1^ Department of Pathology and Translational Genomics, Samsung Medical Center, Sungkyunkwan University School of Medicine, Seoul, Korea; ^2^ Division of Hematology-Oncology, Department of Medicine, Samsung Medical Center, Sungkyunkwan University School of Medicine, Seoul, Korea; ^3^ Department of Health Sciences and Technology, SAIHST, Sungkyunkwan University, Seoul, Korea

**Keywords:** lung cancer, EGFR, KRAS, ALK, molecular epidemiology

## Abstract

**Background:**

*EGFR, KRAS*, and *ALK* alterations are major genetic changes found in non-small cell lung cancers (NSCLCs). Testing advanced lung adenocarcinoma tumors for these three genes is now standard care. The purpose of this study was to investigate the clinicopathologic expression pattern of these three genes in East Asian NSCLC patients.

**Patients and methods:**

We conducted a retrospective study of all patients tested for mutations of these three genes at a single institute in Korea between 2006 and 2014. Study data were extracted from electronic medical records. Univariate and multivariate logistic regression analyses were used to measure associations between clinicopathologic features and alterations of *EGFR*, *KRAS*, and *ALK*.

**Results:**

We detected 12 *EGFR*-mutated tumors with additional mutations in *KRAS* (*N*=6, 0.1%) or *ALK* (*N*=6, 0.1%). General clinicopathologic characteristics of tumors with *EGFR*, *KRAS*, or *ALK* mutations were similar to previous reports. Patients having *EGFR* L858R point mutations were older than patients having *EGFR* exon 19 deletions. *EGFR* G719X point mutations were more common in men and smokers than exon 19 deletions or L858R point mutations. Tumors having *KRAS* G12C mutations were less often of mucinous type than those with G12D or G12V, mutations.

**Conclusions:**

This is the largest three gene molecular epidemiology study in East Asian NSCLC patients. Each genetic alteration was associated with distinct clinicopathologic characteristics. Furthermore, different age and sex are associated with different subtypes of *EGFR* and *KRAS* mutations.

## INTRODUCTION

*EGFR, KRAS*, and *ALK* alterations are the major genetic changes in lung adenocarcinoma[1]. Drugs targeting *EGFR* and *ALK* have improved clinical outcomes in patients with mutations in those genes[2, 3]. Since targeted therapy was discovered, mutation testing has increased[4, 5]. Molecular testing of *EGFR* and *ALK* in lung adenocarcinoma is recommended by the guidelines from College of American Pathologists, the International Association for the Study of Lung Cancer, and the Association for Molecular Pathology[6].

*EGFR* mutation is associated with certain clinical and histologic factors, and is more prevalent in adenocarcinomas, women, Asians, and those who never smoked[7–9]. Despite differences between reports, histology is related to *EGFR* mutation status. Tumors with papillary, micropapillary, acinar, and lepidic (bronchioloalveolar) patterns more frequently have *EGFR* mutations than do tumors with a solid pattern[10–16]. *EGFR* mutation is rare in mucinous adenocarcinoma[17]. *EGFR* mutations tend to occur in older patients[15, 18–21]. Alternatively, *KRAS* mutation is associated with smokers, men, a solid pattern tumors, and mucinous adenocarcinoma[7, 15, 22–24]. *ALK* mutation is associated with non-smokers, younger patients, adenocarcinoma, a solid pattern tumors, and signet ring cell type tumors[25–34].

Genetic alterations of *EGFR*, *KRAS*, and *ALK* typically are mutually exclusive[35]. However, exceptional cases may have concurrent mutations of those genes[36–39]. Sometimes, mutations of other genes can occur after chemotherapy, which can cause resistance to targeted therapy[40–43].

In this study, we characterized the clinicopathologic features and genetic changes associated with *EGFR*, *KRAS*, and *ALK* in lung cancer.

## RESULTS

### EGFR tests

A total of 7,463 *EGFR* mutation tests were performed on samples from 6,878 patients. There were 55 failed tests due to insufficient biopsy materials. Test materials from 254 cases were not from lung cancer. Thus 7,154 tests and 6,583 patients remained (Figure S1). Of these, 545 patients were tested for *EGFR* mutation more than once. Among those patients, 11 had second primary tumors and 1 had a third primary tumor. Among the 6,595 tumors, 2,387 had *EGFR* mutations, and 60 had more than 2 *EGFR* mutations other than T790M.

*EGFR* tests were performed on 4,322 biopsy specimens, 2,548 resected specimens, and 115 cytology specimens. From 4,407 (62.8%) specimens obtained from lung, 4,344 tests were performed by PNA-clamping. Among these, 3,534 tests were confirmed by Sanger sequencing. Sanger sequencing alone was used to test 2,861 tumors. The tumor proportion ranged from 1 to 99% (Table S1). In univariate analysis, the *EGFR* mutation detection rate was low when the specimen was obtained by biopsy (OR[odds ratio]: 0.78, p<0.001), or from lymph node (OR: 0.56, *P*<0.001) or bronchus(OR: 0.67, *P*<0.001), when the tumor proportion was lower than 20% (OR: 0.71, *P*<0.001), or when the test was performed by Sanger sequencing only (OR: 0.81, *P*=0.003). However, in the multivariate analysis, there was no significant difference in mutation rates between biopsy and resection(OR: 1.17, *P*=0.020) or biopsy and cytology (OR: 1.08, *P*=0.874)(Table S2). There was a weak positive correlation between ΔCT-1 and tumor proportion (R^2^ = 0.0068). The ΔCT-1 of T790M was usually less than that of other *EGFR* mutations (Figure S2).

### Association between *EGFR* mutation and clinicopathologic variables

All clinical and histopathologic variables are summarized in Tables S3 and S4. Adenocarcinoma accounted for a large proportion of cases (4,984 cases, 75.6%). The most frequent primary pattern observed was acinar pattern (65.5%). Of the adenocarcinomas, 2,295 (46%) tumors had *EGFR* mutations, 358 (9.2%) had *KRAS* mutations, and 270 (7.2%) had *ALK* rearrangements. 60 tumors (1.2%) had more than 2 *EGFR* mutations other than T790M.

In multivariate analysis, *EGFR* mutations were frequent in women (OR: 1.83, *P*<0.001), middle-aged patients (OR: 1.34, *P*<0.001), those who never smoked (OR: 2.04, *P*<0.001), adenocarcinomas (OR: 14.0, *P*<0.001), well (OR: 2.46, *P*<0.001) to moderately (OR: 2.73, *P*<0.001) differentiated tumors, small-sized tumors (OR for 1cm increase: 0.91, *P*=0.003), tumors of non-mucinous type (OR: 26.8, *P*<0.001), tumors without signet ring cells (OR: 17.2, *P*=0.007), and tumors with lepidic (OR: 2.18, *P*=0.003), acinar (OR: 3.38, *P*<0.001) and papillary (OR: 3.17, *P*<0.001) patterns (Table S5 and Figure S3). The relation between *EGFR* mutation and age was non-linear. In patients under 40, the *EGFR* mutation rate increased with increasing age, while in patients over 60, the *EGFR* mutation rate decreased with increasing age.

### Differences between types of EGFR mutations

Deletions in exon 19 (*N*=1,262) and L858R point mutations (*N*=921) were the most common mutations. These two mutations accounted for approximately 90% of all *EGFR* mutations. Less common mutations included G719X point mutations (*N*=81), insertions in exon 20 (*N*=54), S768I point mutations (*N*=20), insertions in exon 19 (*N*=11), and L861Q point mutations (*N*=10) (Table S6).

**Figure 1:**
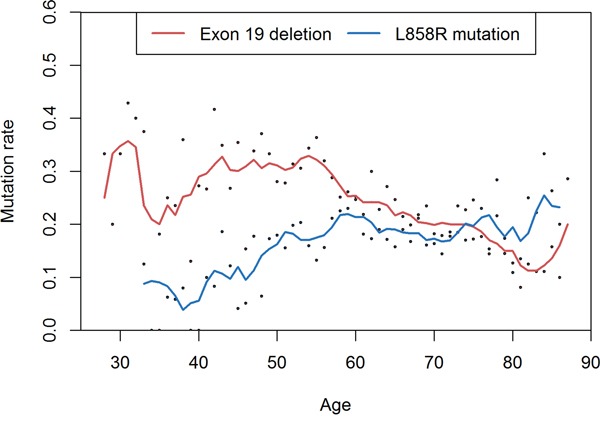
Comparison between exon 19 deletion and L858R point mutation Deletions in exon 19 are frequent in younger patients and L858R mutations are frequent in older ages.

**Figure 2:**
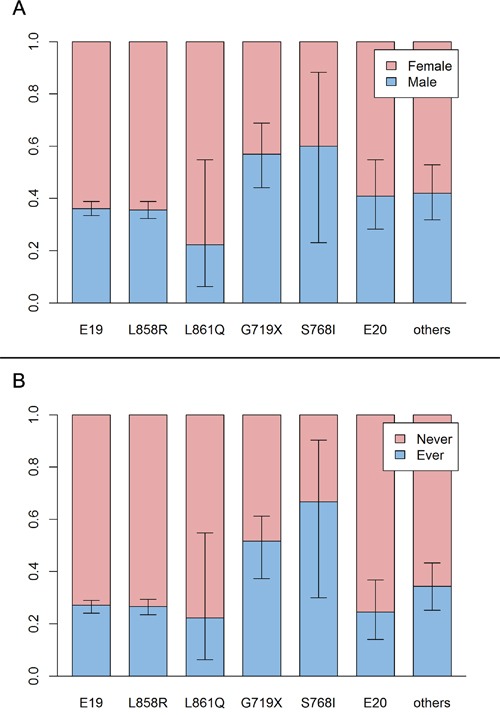
The proportion and subtypes of EGFR mutation **A.** Sex proportion and subtypes of EGFR mutation. Deletion in exon 19 and L858R appears more often in women. However, G719X and S768I do not have this tendency. **B.** Proportion of smokers and subtypes of EGFR mutation. The trend is similar to the sex proportion. E19: deletion in exon 19, E20: insertion in exon 20.

Deletions in exon 19 frequently occurred in younger patients (OR for 1-year increase: 0.98, *P*<0.001). Conversely, L858R point mutations frequently occurred in older patients (OR for 1-year increase: 1.02, *P*<0.001). In multivariate analysis comparing *EGFR* mutation types, older patients were more likely to have L858R mutations than exon 19 deletions (OR for 1-year increase: 1.03, *P*<0.001) (Figure 1). Compared to exon 19 deletion, G719X mutation was more likely to occur in men(OR: 1.69, *P*=0.167) and smokers (OR: 2.04, *P*=0.058), but those factors were not independent in multivariate analysis (Table 1 and Figure 2).

**Table 1:** Multivariate analysis of subtypes of *EGFR* mutation

vs. E19		Age (per 1 year)		Sex (male vs. female)		Smoking (ever vs. never)	
		OR	*P*-value	OR	*P*-value	OR	*P*-value
E19 vs.	L858R	1.03	<0.001	1.01	0.914	1.00	0.994
	L861Q	1.07	0.050	0.38	0.389	1.68	0.645
	G719X	1.03	0.028	1.69	0.167^a^	2.04	0.058^a^
	S768I	0.97	0.418	1.19	0.889	4.59	0.227
	E20	1.00	0.756	1.66	0.178	0.61	0.247

a p-value is less than 0.001 in univariate analysis

E19: exon 19 deletion, E20: exon 20 insertion

### Primary T790M mutation

There were 15 patients with a T790M *EGFR* mutation without history of previous targeted therapy (primary T790M mutation). One primary T790M mutation presented without other *EGFR* mutations. Eight of these patients were women and nine had never smoked. Their mean age was 65.3 years, and all patients had adenocarcinoma. The ΔCT-1 of secondary (patients who received targeted therapy) T790M was lower than the ΔCT-1 of coexisting *EGFR* mutations (average difference of ΔCT-1: 2.74). However, the ΔCT-1 of the primary T790M mutation was not very different from the ΔCT-1 of coexisting *EGFR* mutations (average difference of ΔCT-1: −0.20). Ten patients were treated with *EGFR* inhibitors. Tumor progressed in nine patients, while insufficient time has passed to assess the other patient (Table 2).

**Table 2:** Clinical Data of Patients Having Primary T790M mutation

	Age	Sex	Smoking	Other EGFR mutation	ΔCT-1(other than T790M)	ΔCT-1 (T790M)	Targeted therapy	Response
PT01	53	M	Former	Positive	3.9	4.59		
PT02	57	F	Former	Positive			Gefitinib	PD
PT03	70	F	Former	Positive			Gefitinib	PD
PT04	63	F	Never	Positive			Gefitinib, Lapatinib	PD
PT05	83	F	Never	Positive				
PT06	78	M	Never	Negative				
PT07	57	F	Never	Positive			Gefitinib	PD
PT08	65	M	Former	Positive			Gefitinib, Afatinib	PD
PT09	53	M	Never	Positive	8.06	6.72		
PT10	41	M	Former	Positive	5.99	5.74	Gefitinib	PD
PT11	78	M	Former	Positive	2.75	4.17	Gefitinib	PD
PT12	77	F	Never	Positive	4.46	4.63	Gefitinib	PD
PT13	69	F	Never	Positive	3.7	3.89		
PT14	75	M	Never	Positive	4.91	5.64	Gefitinib	NA
PT15	61	F	Never	Positive	6.31	6.29		

PD: progressed disease, NA: not accessible due to short follow up time

### Association between *KRAS* mutation and clinicopathologic variables

**Figure 3:**
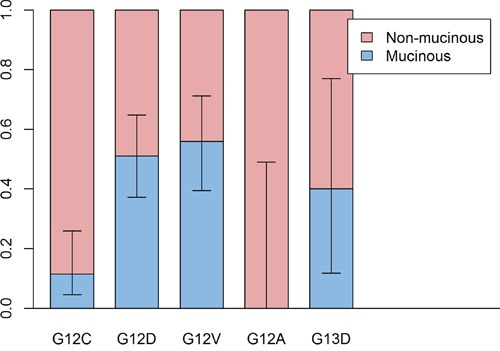
Comparison of proportion of mucinous type between subtypes of KRAS mutation Proportion of mucinous type is higher in G12D and G12V subtypes than G12C subtype.

In multivariate analysis, *KRAS* mutations were frequent in men (OR: 1.67, *P*=0.003), older patients (OR for 1-year increase: 1.03, *P*<0.001), smokers (OR: 1.78, *P*<0.001), adenocarcinomas (OR: 7.28, *P*<0.001), large-sized tumors (OR for 1cm increase: 1.17, *P*<0.001), poorly-differentiated tumors (vs. moderate differentiation, OR: 1.88, *P*=0.001), and mucinous type (OR: 9.09, *P*<0.001) and solid pattern (vs. acinar pattern, OR: 2.57, *P*<0.001) tumors (Table S7). Among those variables, mucinous type was the most distinguishing factor. There were three prevalent *KRAS* mutations: G12C (*N*=108, 27.2%), G12D (*N*=107, 27.0%), and G12V (*N*=89, 22.3%). G12C mutations were infrequent in mucinous type tumors compared to G12D (OR: 4.98, *P*=0.007) and G12V mutations (OR; 5.58, *P*=0.006) (Figure 3). In univariate analysis, G12C mutations were frequent in men and smokers compared to G12D and G12V mutations. However, those were not independent factors in multivariate analysis (Table 3 and Figure S4).

**Table 3:** Multivariate analysis of subtypes of *KRAS* mutations

		Non-mucinous vs. Mucinous		Sex (Male vs. Female)		Smoking (Never vs. Ever)	
		OR	P-value	OR	P-value	OR	P-value
vs. G12C	G12D	4.98	0.007	0.96	0.968^a^	0.18	0.044^a^
	G12V	5.58	0.006	1.02	0.988^a^	0.18	0.060^a^
	G12A	0.73	0.841	0.43	0.788	0.38	0.755
	G13D	7.22	0.082	1.04	0.983	0.85	0.945

a p-value is less than 0.01 in univariate analysis

### Association between *ALK* rearrangement and clinicopathologic variables

In multivariate analysis, *ALK* rearrangements were frequent in younger patients (OR for 1 year increase: 0.95, *P*<0.001), those who never smoked (OR: 1.73, *P*=0.005), adenocarcinomas (OR: 6.99, *P*<0.001), poorly differentiated tumors (vs. moderate differentiation, OR: 2.54, *P*<0.001), signet ring cell types (OR: 20.3, *P*<0.001), cribriform (vs. acinar pattern, OR: 22.9, p<0.001) or solid patterns (vs. acinar pattern, OR: 2.96, *P*=0.002), tumors with lymph node metastasis (N2 vs N0, OR: 3.95, *P*<0.001), and tumors invading blood vessels (OR: 3.85, *P*<0.001), lymphatic vessels (OR: 2.13, *P*=0.004), or nerves (OR: 2.96, *P*=0.019) (Table S8 and Figure S5). Signet ring cell type and cribriform pattern were highly associated with *ALK* rearrangements.

### Double mutations

Among tumors with *EGFR* mutations, 12 had additional mutations in *KRAS* (*N*= 6) or *ALK* (*N*=6) (Figure S6). Three of these *EGFR* mutations were detected only by PNA clamping and not by Sanger sequencing. Nine of these *EGFR* mutations were confirmed by Sanger sequencing, seven of which were major subtypes of *EGFR* mutation (deletion in exon 19 and L858R point mutation). Four were G719X point mutations, comprising 33% of the double mutants, which is a higher proportion than that observed in tumors having only *EGFR* mutations. The other *EGFR* mutation was an R803W point mutation, a very rare subtype. In five tumors, *KRAS* mutations presented at codon 12 or 13, and one tumor had two *KRAS* mutations, at codons 21 and 34. Among the six *ALK* alterations tested by immunohistochemistry, two were confirmed by FISH. The mean age of patients having both *EGFR* and *ALK* mutations was higher than that of patients having *ALK* rearrangements (*P*=0.012). Except for one tumor, tumors having both *EGFR* and *KRAS* mutations were moderately differentiated. Tumors having both *EGFR* and *ALK* mutation tended to be poorly differentiated. Nine of 15 patients had stages higher than III. One patient had a history of *EGFR* targeted therapy and ALK targeted therapy prior to mutation testing. The remaining patients had no history of targeted therapy prior to mutation tests. Six patients were treated with *EGFR* tyrosine kinase inhibitors and three were treated with ALK inhibitors. The follow-up period was insufficient to measure response (Table 4).

**Table 4:** Patients having *EGFR* mutation plus *KRAS* or *ALK* mutations

	Age	Sex	Smoking	Pack-year	Stage	*EGFR* mutation	*KRAS* mutation	Differentiation	Targeted therapy
DM01	77	M	Current	57	IIA	L858R	I21S, P34S	Moderate	
DM02	79	F	Never		IV	G719X	G12D	Moderate	
DM03	51	F	Never		IIIA	Exon 19 deletion	G12V	Moderate	erlotinib
DM04	64	F	Never		IV	L858R	G12D	Unknown	gefitinib
DM05	64	F	Never		IA	L858R	G13A	Moderate	
DM06	58	M	Former	20	IIIA	exon 19 deletion	G13C	Poor	gefitinib
	**Age**	**Sex**	**Smoking**	**Pack-year**	**Stage**	***EGFR* mutation**	**ALK methods**	**Differentiation**	**Targeted therapy**
DM07	63	F	Never		IIB	L858R	IHC	Poor	gefitinib
DM08	67	F	Never		IV	R803W	IHC	Poor	erlotinib
DM09	69	F	Never		IIIA	G719X	IHC	Moderate	
DM10	57	M	Former	15	IV	G719X	IHC	Poor	crizotinib
DM11	59	F	Never		IV	Exon 19 deletion	IHC & FISH	Unknown	crizotinib
DM12	63	F	Never		IV	G719X	IHC & FISH	Moderate	gefitinib

M: male, F: female, IHC: immunohistochemistry, FISH: fluorescence in situ hybridization

### Double primary tumors

Among the 12 identified second or third primary tumors, 10 had genetic profiles that differed from their previous tumors. The histologic type was different in one second primary tumor. Another second primary tumor was histologically similar to the previous tumor, and had no mutations in *EGFR*, *KRAS*, or *ALK*. All second primary tumors arose at different sites from the prior tumors (Table S9).

## DISCUSSION

We analyzed data from a large number of lung cancer patients from a single institution, assessing genetic alterations of *EGFR*, *KRAS*, and *ALK*. Most results were consistent with previous reports[7, 10, 16, 48]. However, contrary to previous reports[18, 19], *EGFR* mutations were more frequent in tumors from patients between 40 and 64 years of age than from other age groups. The relationship between age and *EGFR* mutation frequency was different with different mutation type. Exon 19 deletions occurred frequently in patients under 65, while L858R point mutations occurred frequently in patients over 40. Summing these data, the *EGFR* mutation frequency was highest in middle-aged patients. One report describes similar comparison of age between *EGFR* mutation subtypes[7]. Although it did not reach statistical significance in multivariate analysis, the G719X point mutation was frequent in men and smokers than other mutation subtypes. Of the 81 patients with G719X mutations, 44 (54%) were men and 39 (48%) smoked. This finding is similar to a previous report[39].

The T790M mutation is the most common cause of *EGFR*-targeted therapy resistance[49]. This mutation typically is detected after targeted therapy and is present as a minor clone prior to treatment[50]. In the 15 cases with primary T790M mutations here, the average difference in ΔCT-1 between T790M and other coexisting *EGFR* mutations was −0.20, whereas the average difference between T790M and other coexisting *EGFR* mutations was 2.73 in secondary T790M mutations. The ΔCT-1 of primary T790M was not very different from the ΔCT-1 of other coexisting *EGFR* mutations, indicating that the T790M mutation was present as a major clone in these cases. The T790M mutation may play an important role in this situation other than just resistance to *EGFR* tyrosine kinase inhibitors. There was no clinicopathologic difference in our analysis between patients with primary T790M mutations and patients without primary T790M mutations. A recent study with more patients with primary T790M mutations showed that primary T790M mutation is associated with never smoking and development of brain metastasis[51].

*KRAS* mutations were frequent in men, older patients, smokers, adenocarcinomas, mucinous tumor types, large-sized tumors, poorly differentiated tumors, and tumors with a solid pattern, consistent with previous reports[23, 24]. *ALK* rearrangements were frequent in younger patients, those who never smoked, adenocarcinomas, poorly differentiated tumors, signet ring cell types, and tumors with cribriform or solid patterns, also consistent with previous reports[33, 47]. All *KRAS* mutations were point mutations. Like the L858R point mutation of *EGFR*, the *KRAS* mutation rate increased as patient age increased. All *ALK* mutations were chromosomal rearrangements. Like *ALK* rearrangements in other tumors[52, 53], *ALK* rearrangements in lung cancer frequently occur in younger patients. G13C mutations were infrequent in mucinous types compared with G12D and G12V point mutations. According to another report, G12C is associated with smokers and G12D is associated with never smoking[7]. However, in our data, smoking was not an independent factor in multivariate analysis.

Generally, *EGFR*, *KRAS*, and *ALK* mutations are mutually exclusive. There are few reports of lung cancer with concurrent mutations of these genes[36–39]. In many of these, the secondary mutation was not detected at diagnosis, but after targeted therapy. These secondary mutations in other genes can promote resistance to targeted therapy. We identified 12 tumors (0.2%) having an *EGFR* mutation and an additional *KRAS* or *ALK* mutation. Only one patient had received prior targeted therapy. Of the 12 *EGFR* mutations, 7 were of a common type (exon 19 deletion and L858R point mutation), 4 were G719X point mutations, and 1 was a R803W point mutation. The proportion of rare mutations like the G719X point mutation was high in these tumors. The rare S768I point mutation was identified frequently in another study[39]. Intratumoral heterogeneity has been reported in lung cancer having both *EGFR* and *ALK* alterations[54]. Here, 9 of 12 cases were higher than stage III. It is likely that a second mutation occurred during tumor progression.

Twelve second or third primary tumors were included in this study. Among them, 10 had distinct genetic changes from the prior tumors. A second or third primary tumor is not uncommon in lung cancer. Distinguishing a second primary tumor from recurrence by clinical features or histologic features can be difficult, though genetic profiling can be helpful. If the genetic alteration differs from the prior tumor, this identifies the second as another primary tumor[55].

*EGFR* test results are influenced by several factors. When tissue was obtained from lymph nodes or bronchus, the *EGFR* mutation rate was lower (odds ratio: 0.56 and 0.67 each). It can be concluded that *EGFR* tests done with lymph node or bronchus specimens have a one-third false negative rate. Since the lymph node and bronchus usually are biopsied by bronchoscopy, the tissue sample is small. Dense lymphocytes in lymph nodes also dilute tumor DNA. These facters make the tests less sensitive. The *EGFR* mutation rate did not differ between tissues obtained from bone or body fluid. Tumor proportion was also important. When tumor proportion was below 20%, the *EGFR* mutation rate decreased. When tumor proportion was below 5%, the *EGFR* mutation was detected less than half as often. To make accurate tests, tumor proportion must be above the analytical sensitivity of the testing method. When the tumor proportion is low, a more sensitive method should be used[56].

Since our data were extracted from past medical records, some data were missing, and the data may contain inaccuracies. The number of cases was large enough to measure detailed trends of association between clinicopathologic features and genetic alterations of *EGFR*, *KRAS*, and *ALK*. *EGFR* exon 19 deletions and L858R point mutations tend to occur at different ages. The *EGFR* G719X point mutation differs from other subtypes in that age and sex are equal, and G719X commonly coexists with another gene mutation. The *KRAS* G12C point mutation was less frequently associated with mucinous type. However, more cases are required to characterize other rare subtypes of *EGFR* and *KRAS* mutations.

In this study, we analyzed the clinicopathologic features associated with three major driving mutations of lung cancer. Each subtype of driving mutation will occur by different mechanisms of mutagenesis in a different environment which is related to age, sex, and smoking history. The driving mutation and related risk factors are associated with morphology and behavior of the tumor. These data are valuable in understanding the characteristics of lung cancer.

## MATERIALS AND METHODS

### Study design

We conducted a retrospective study of all patients whose tumors were tested for *EGFR*, *KRAS*, and *ALK* mutation at the Samsung Medical Center (Seoul, Korea) from 2006 to 2014. The study was approved by the Institutional Review Board of the Samsung Medical Center. The requirement for informed consent was waived, as the study was based on existing data.

### Data collection

Study data were automatically or manually extracted from electronic medical records. Clinical data included sex, age when testing was performed, smoking history, origin of cancer, and *EGFR, KRAS* and *ALK* mutation status. Data regarding *EGFR* testing methods included biopsy methods, organs biopsied, tumor proportion of material sampled, test methods, report date, ΔCT-1(the difference in CT value between the negative control and test sample[44]) and test results including the type of *EGFR* mutation. Pathologic data included tumor type, histologic pattern, tumor size, pathologic stage, and the presence of lymphatic, vascular, or pleural invasion. All pathologic data except type of tumor refers only to resected tumors. Histologic pattern was assessed only for adenocarcinoma. When an *EGFR* mutation was identified together with a *KRAS* or *ALK* mutation, the tissue slide and chromatogram of Sanger sequencing were reviewed.

### Detection of alterations of *EGFR, KRAS* and *ALK*

*EGFR* gene alteration was detected by either real-time PCR with PNA-clamping methods, direct sequencing, or both methods. The PNA-Clamp™*EGFR* Mutation Detection kit (PANAGENE, Inc., Daejeon, Korea) was used for real-time PCR, performed as described[45]. When detection was done only with direct sequencing, exon 18, 19, 20, and 21 were sequenced as previously described[44]. When both methods were used, exons containing mutations detected by real-time PCR were sequenced, and exon 19 was sequenced if no mutation was detected by real-time PCR.

*KRAS* gene alteration was also detected by either real-time PCR with PNA-clamping methods, direct sequencing, or both methods. The PNA-Clamp™KRAS Mutation Detection kit (PANAGENE, Inc., Daejeon, Korea) was used for real-time PCR, performed as described[46]. KRAS exon 2, which contains codons 12 and 13, was sequenced by direct sequencing as previously described[44].

*ALK* gene alteration was detected by immunohistochemistry or fluorescence in situ hybridization(FISH)[47].

### Statistical analysis

We used means and standard deviations to summarize continuous variables and counts and numbers with percentages to summarize categorical variables. Age was categorized into three groups: group 1, younger than 40 years; group 2, between 40 and 64 years; group 3, older than 64 years. Univariate and multivariate logistic regression analyses were used to determine the association between each variable and *EGFR*, *KRAS* and *ALK* mutations. Differences between subtypes of *EGFR* and *KRAS* mutations were tested using multinomial logistic regression. *P*-values of less than 0.01 were considered statistically significant.

## SUPPLEMENTARY FIGURES AND TABLES


