# Supplementary material for: Clinicopathologic characteristics of EGFR, KRAS, and ALK alterations in 6,595 lung cancers
Source: Oncotarget. 2016 Mar 14;7(17):23874–84. doi: 10.18632/oncotarget.8074 (PMC5029670; doi:10.18632/oncotarget.8074)
Supplement: Supplementary file 1 [file oncotarget-07-23874-s001.pdf]

Clinicopathologic characteristics of *EGFR*, *KRAS*, and *ALK* alterations in 6,595 lung cancers

SUPPLEMENTARY FIGURES AND TABLES

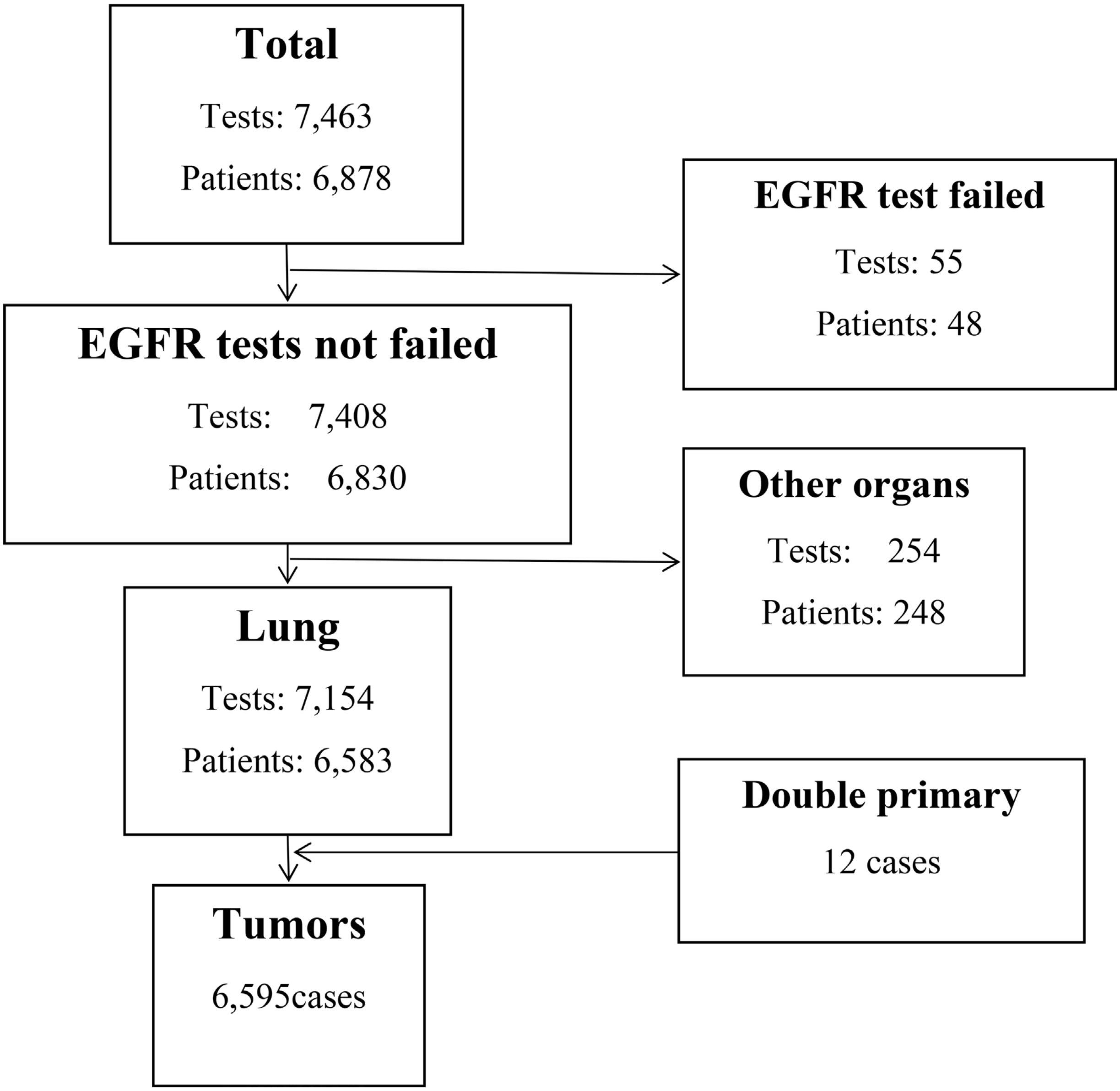

Supplementary Figure S1: Number of tests and patients being analyzed. A total of 7,154 *EGFR* tests and 6,595 tumors were analyzed.

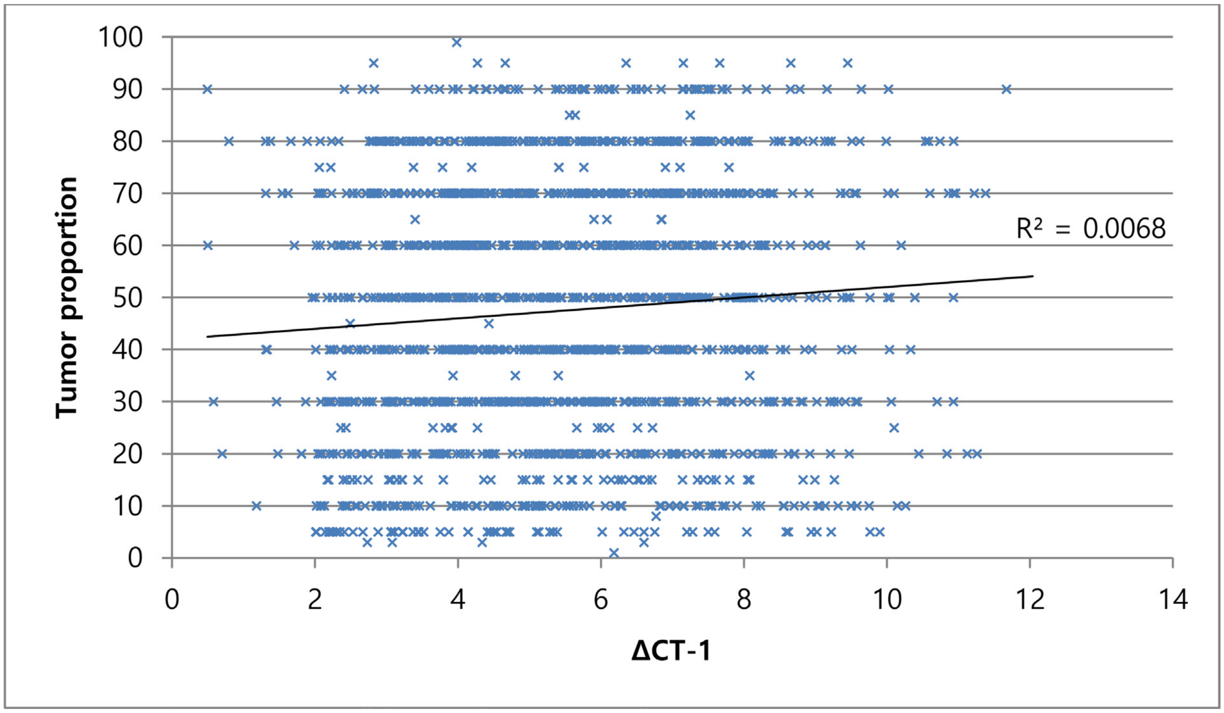

Supplementary Figure S2a: Relationship between ΔCT-1 and tumor proportion in the test specimen. There is a weak positive correlation between ΔCT-1 and tumor proportion.

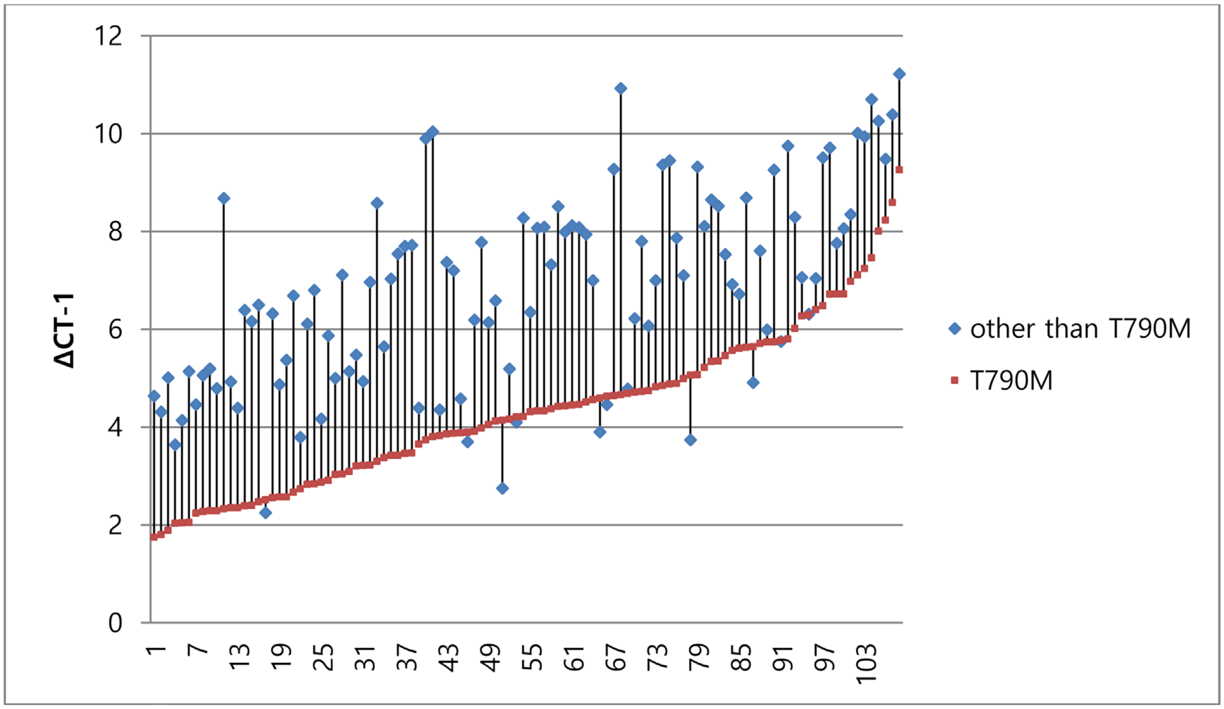

Supplementary Figure S2b: ΔCT-1 of T790M and coexisting *EGFR* mutations. ΔCT-1 of T790M is generally lower than that of coexisting *EGFR* mutations

Supplementary Figure S3: Association between clinicopathologic features and *EGFR* mutation.

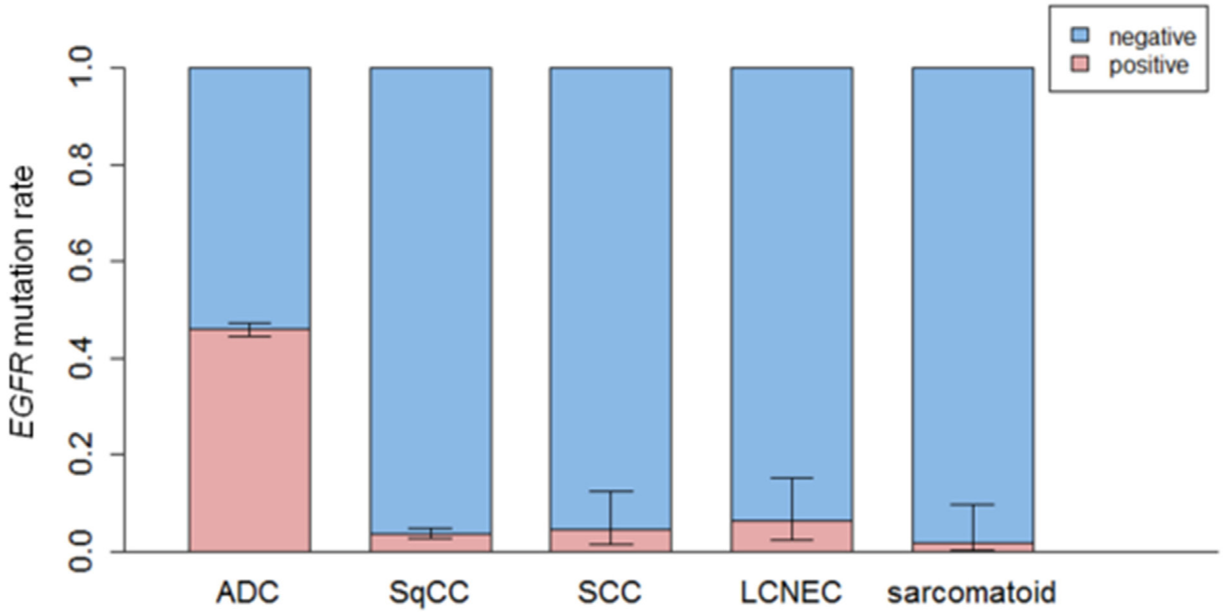

Supplementary Figure S3a: Histologic types of lung cancer and *EGFR* mutation rate. *EGFR* mutation is mostly present in adenocarcinoma. Other histologic types of tumor also have small percentage of *EGFR* mutation.

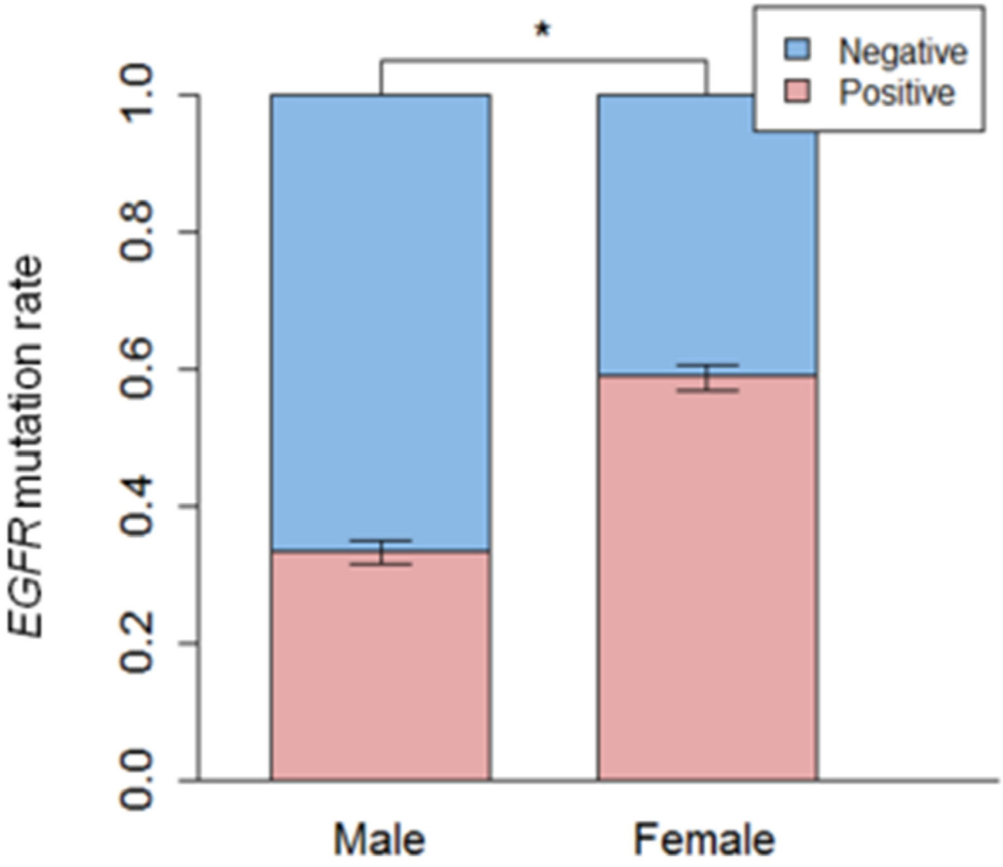

Supplementary Figure S3b: Sex and *EGFR* mutation rate. *EGFR* mutations are frequent in female patient

(*Continued*)

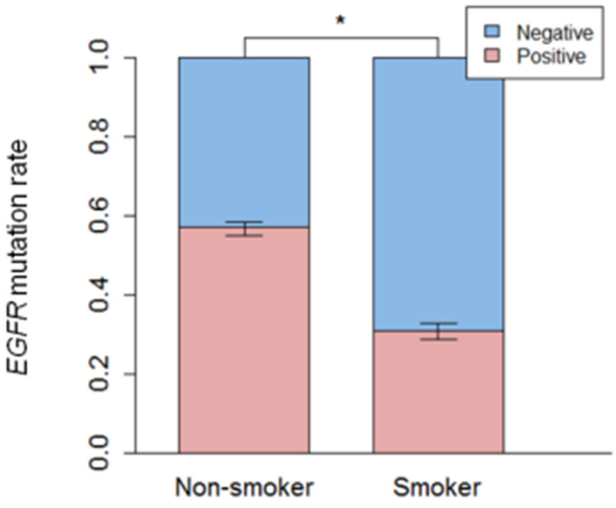

Supplementary Figure S3c: Smoking history and *EGFR* mutation rate. *EGFR* mutations are frequent in non-smoker.

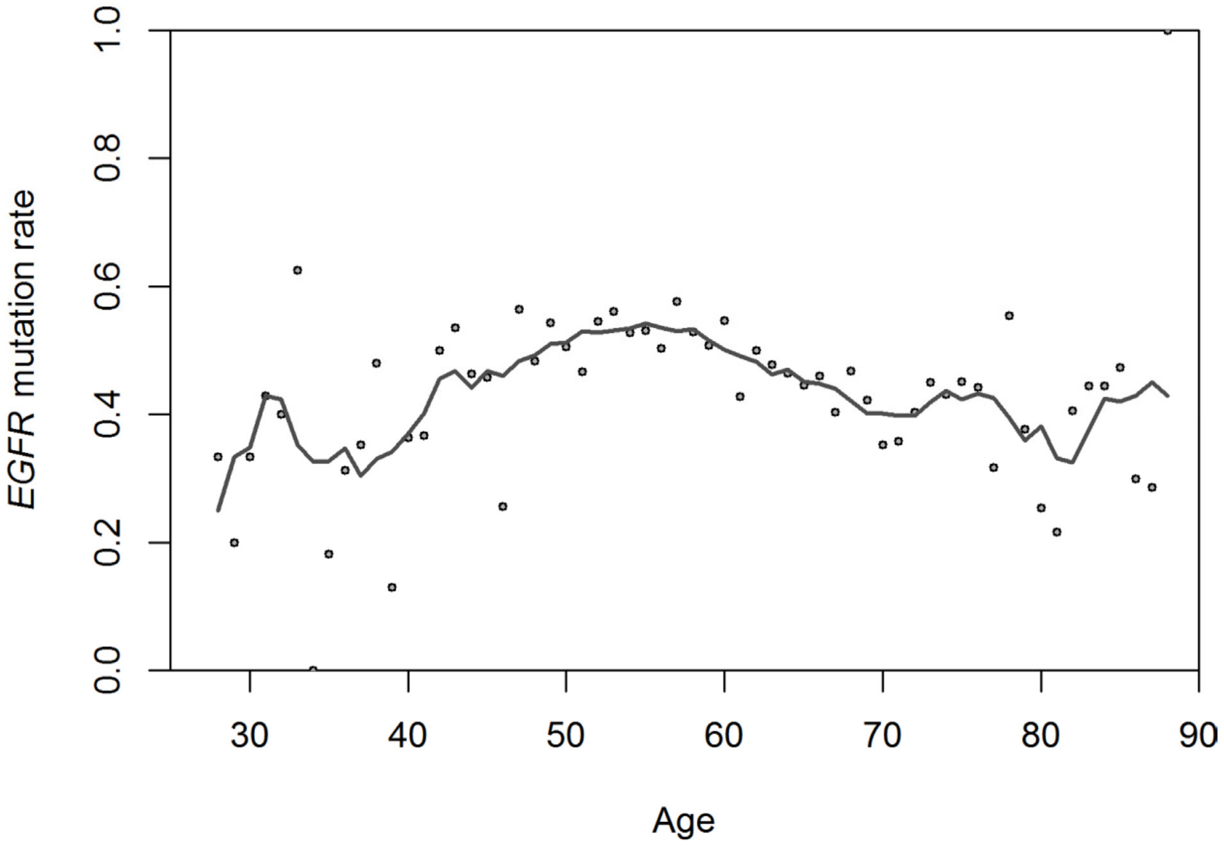

Supplementary Figure S3d: Age of patients and *EGFR* mutation rate. Dots represent *EGFR* mutation proportion at specific ages. The line represents the moving average trend of *EGFR* mutation proportion. The proportion of *EGFR* mutation is highest between 40 and 65 years of age.

(*Continued*)

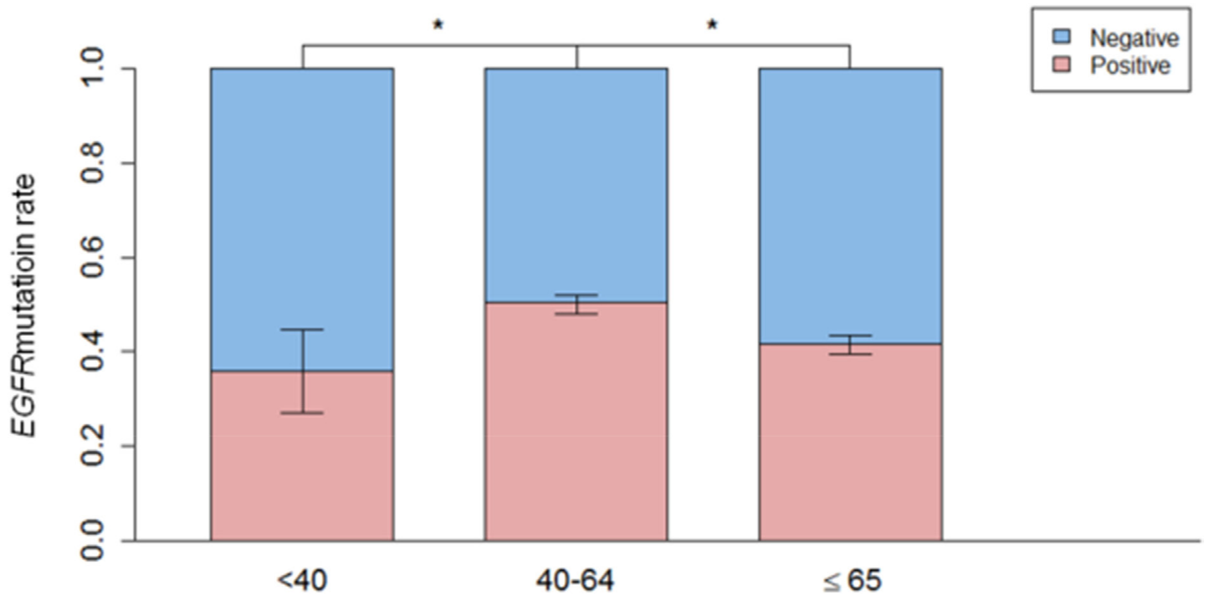

Supplementary Figure S3e: Age group and *EGFR* mutation rate. EGFR mutations are frequent in age between 40 and 64.

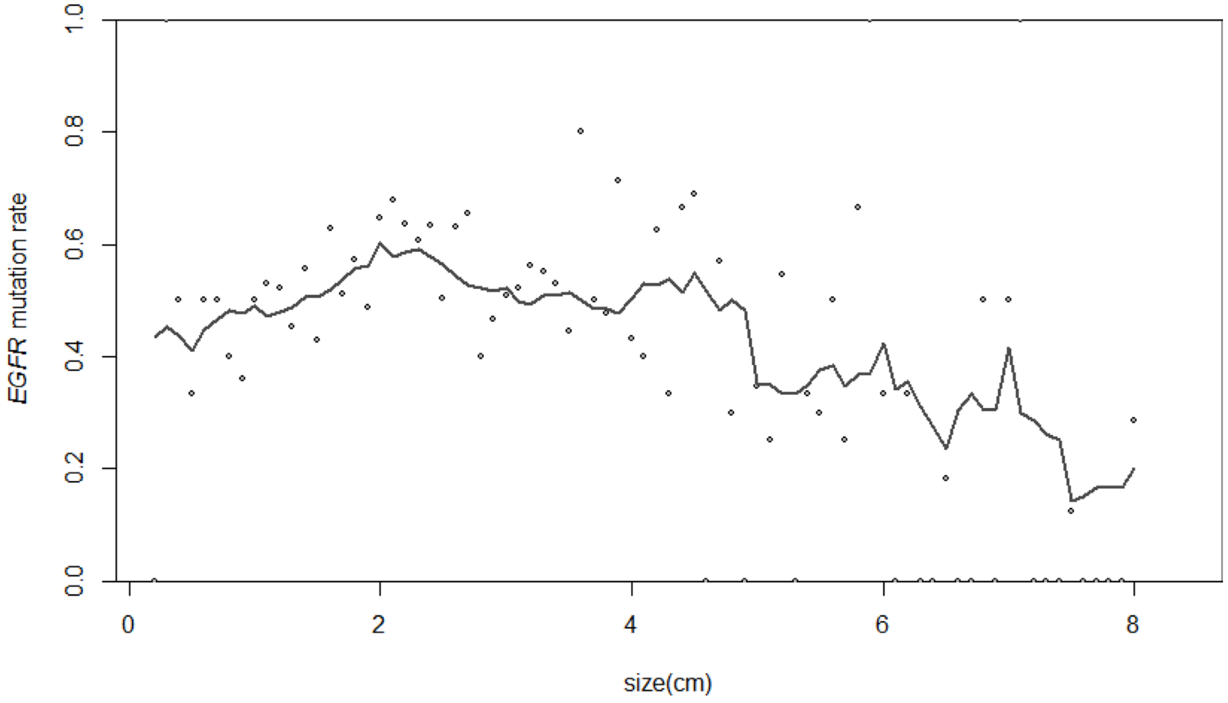

Supplementary Figure S3f: Tumor size and *EGFR* mutation rate. Dot represents proportion of *EGFR* mutation at certain age. Line represents moving average trend line of *EGFR* mutation proportion. EGFR mutations are less frequent in large-sized tumor.

(*Continued*)

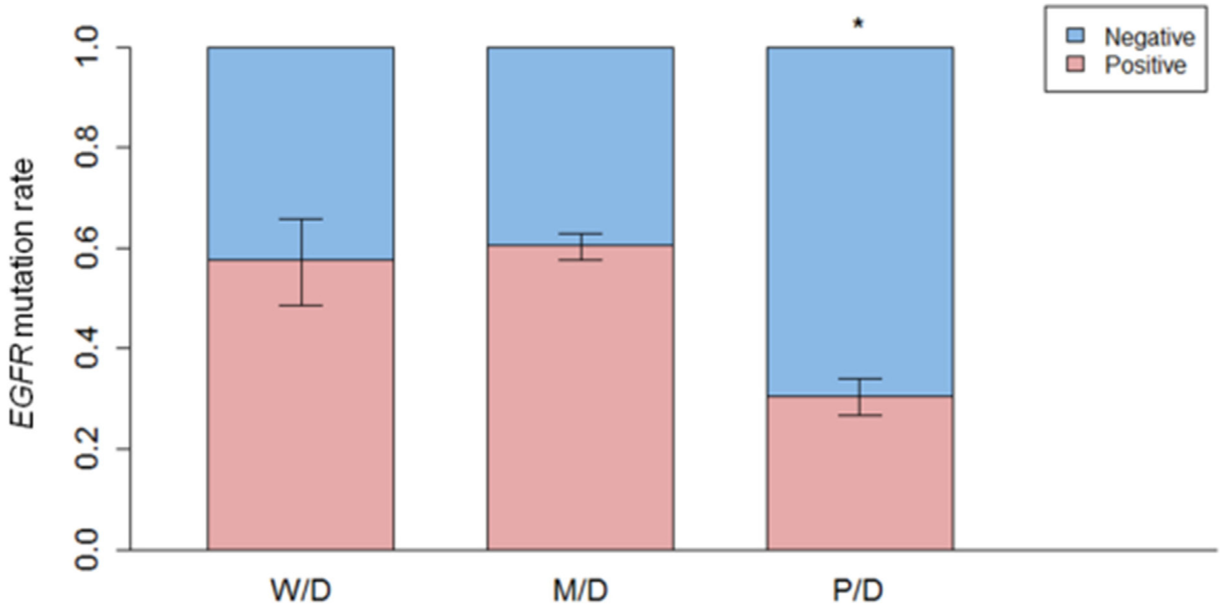

Supplementary Figure S3g: Tumor differentiation and *EGFR* mutation rate. *EGFR* mutations are less frequent in poorly differentiated tumor.

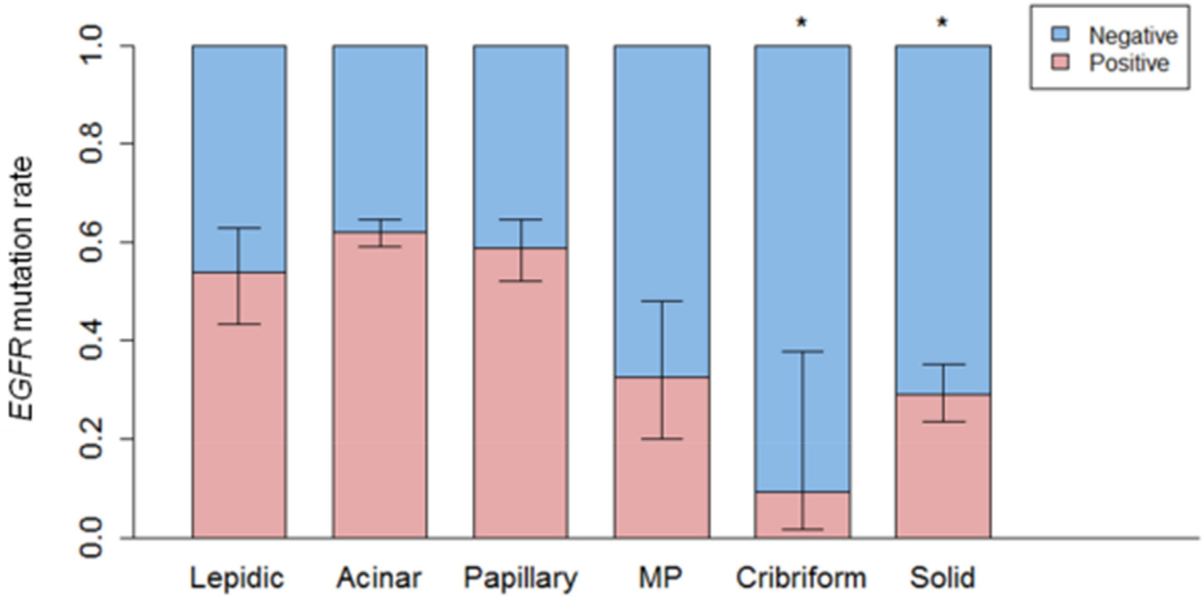

Supplementary Figure S3h: Primary pattern and *EGFR* mutation rate. *EGFR* mutations are less frequent in cribriform or solid pattern.

(*Continued*)

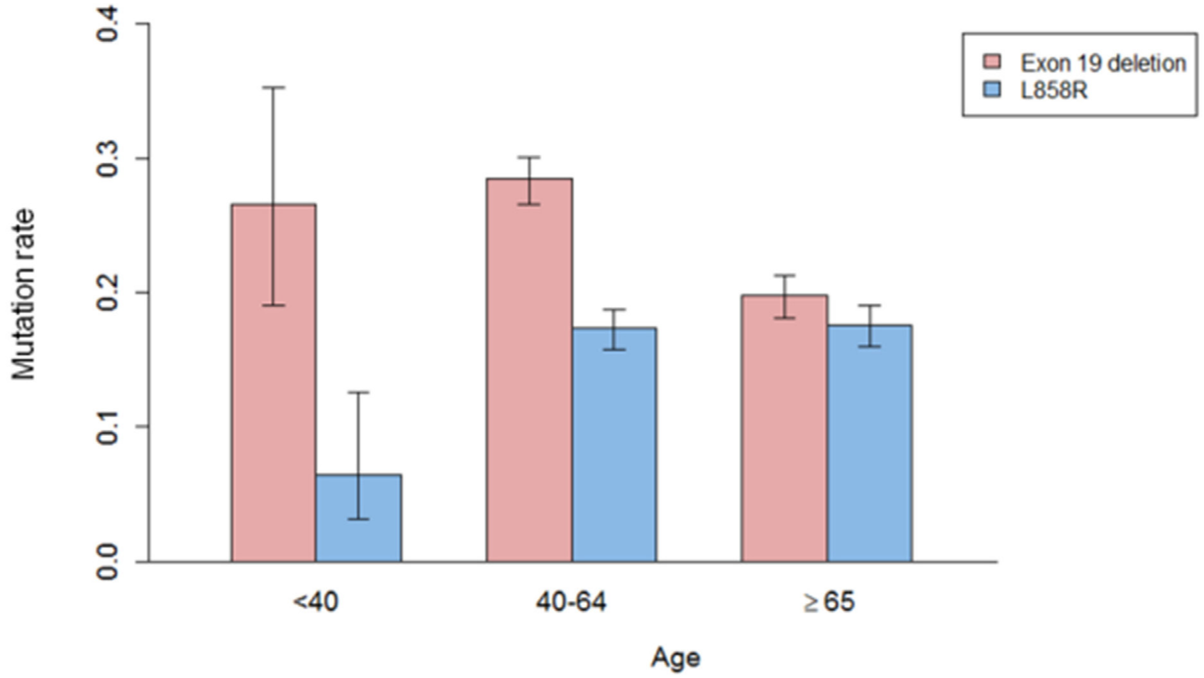

Supplementary Figure S3i: Age group and subtypes of *EGFR* mutation rate. Exon 19 deletions are frequent in age less than 65 years. L858R mutations are frequent in age more than 39 years.

Supplementary Figure S4: Association between clinicopathologic features and *KRAS* mutation.

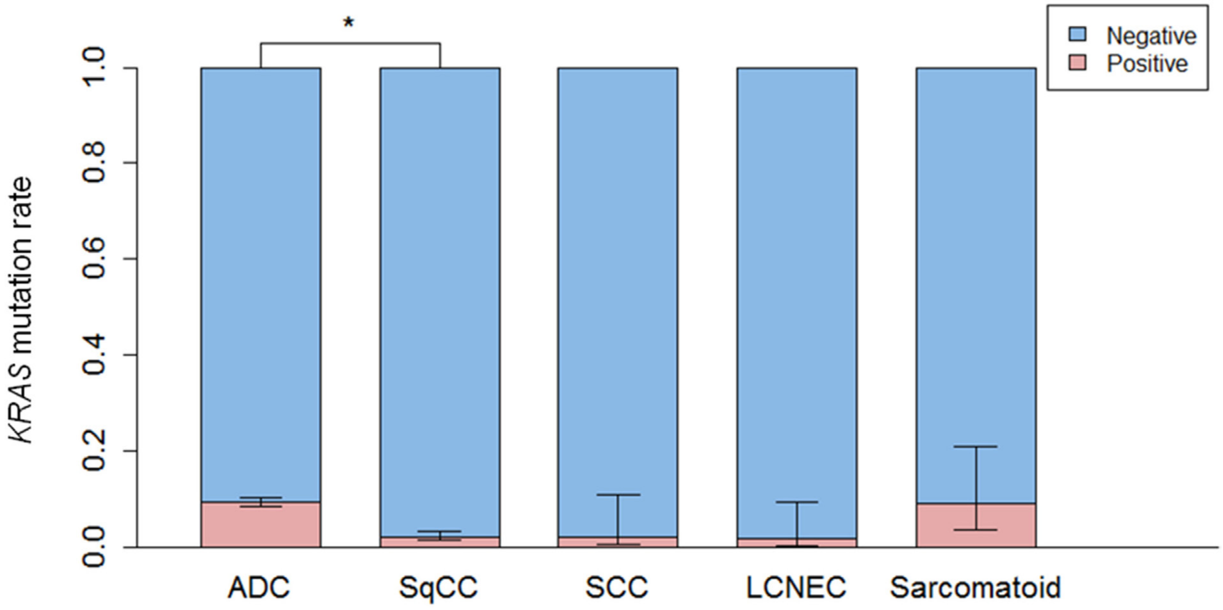

Supplementary Figure S4a: Histologic types of lung cancer and *KRAS* mutation rate. *KRAS* mutations are frequent in adenocarcinoma.

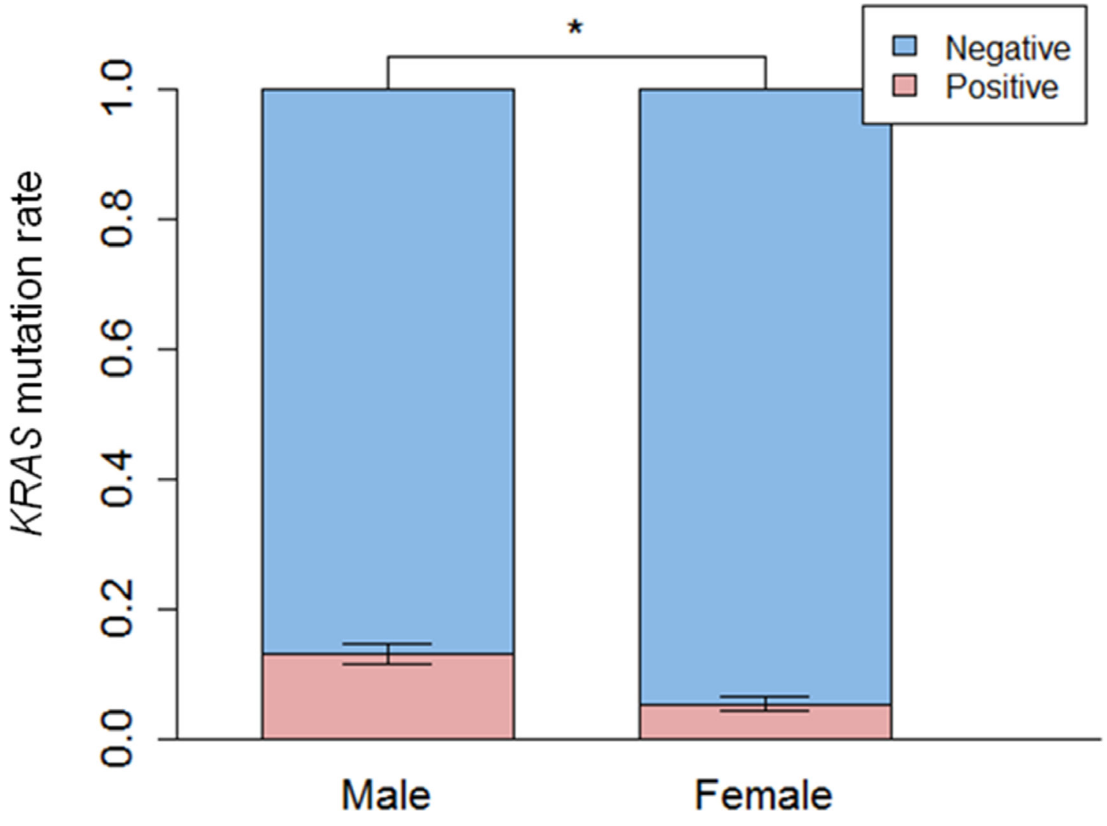

Supplementary Figure S4b: Sex and *KRAS* mutation rate. *KRAS* mutations are frequent in male patient.

(*Continued*)

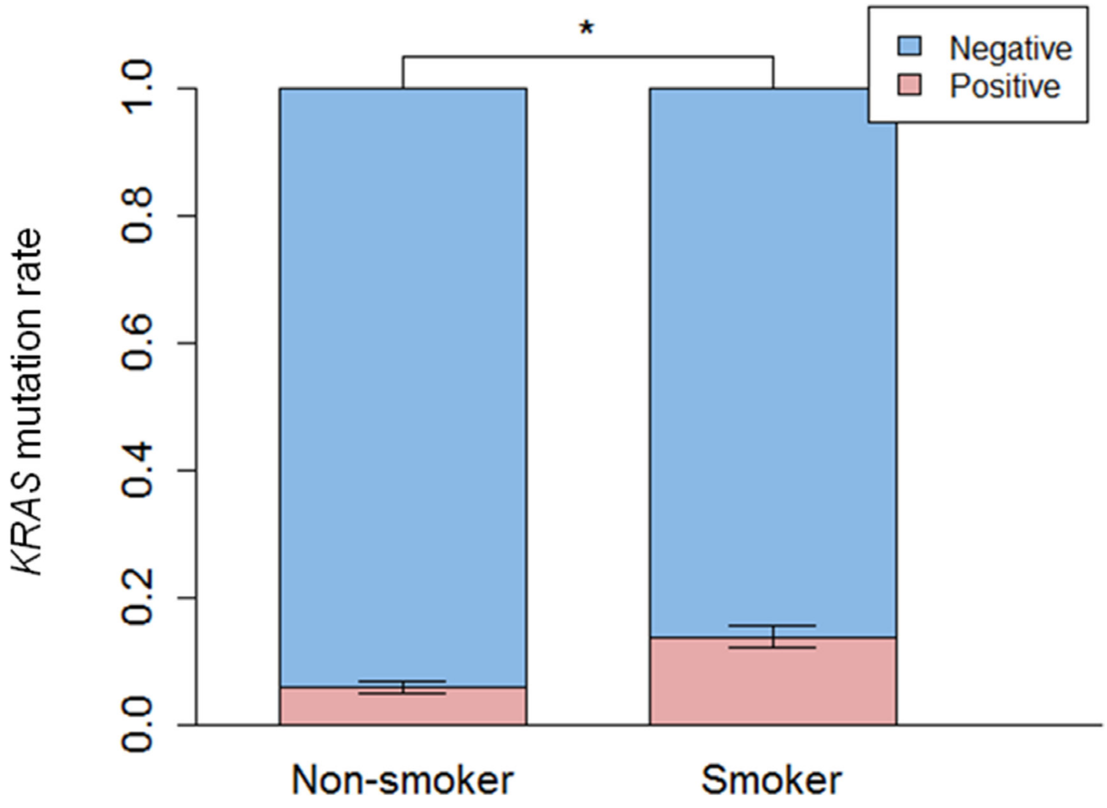

Supplementary Figure S4c: Smoking history and *KRAS* mutation rate. *KRAS* mutations are frequent in smoker.

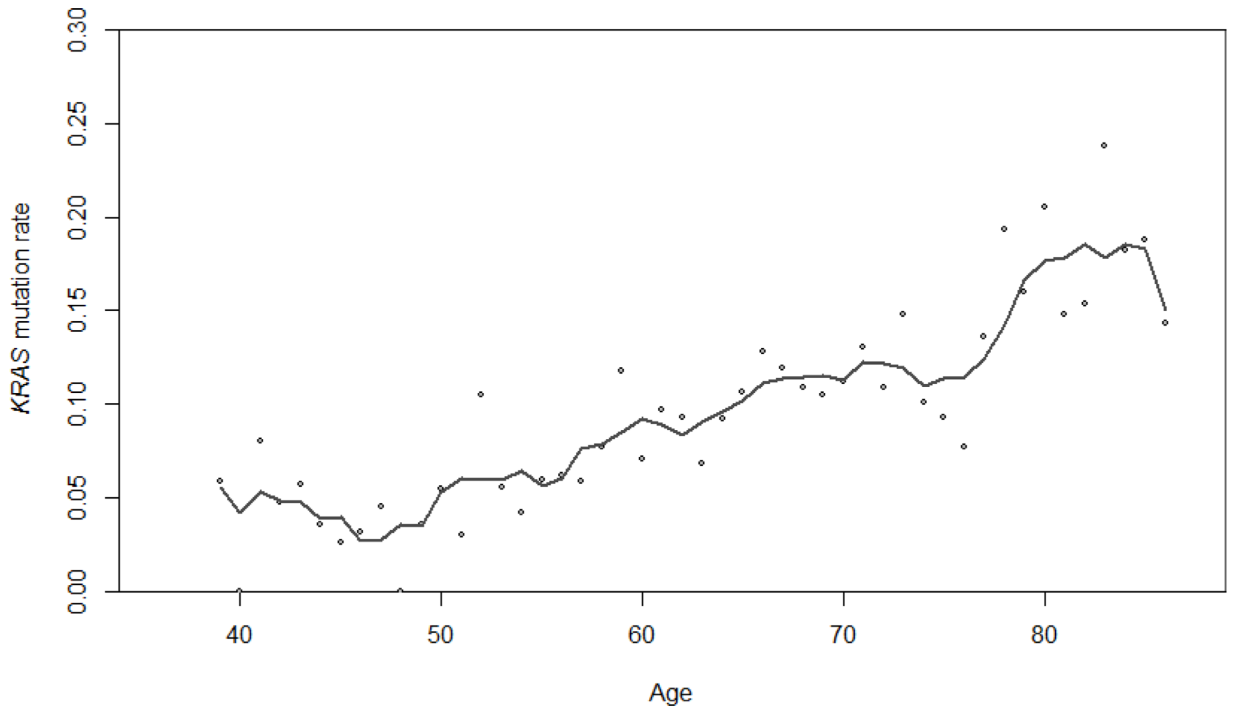

Supplementary Figure S4d: Age and *KRAS* mutation rate. *KRAS* mutations are frequent in older patient.

(*Continued*)

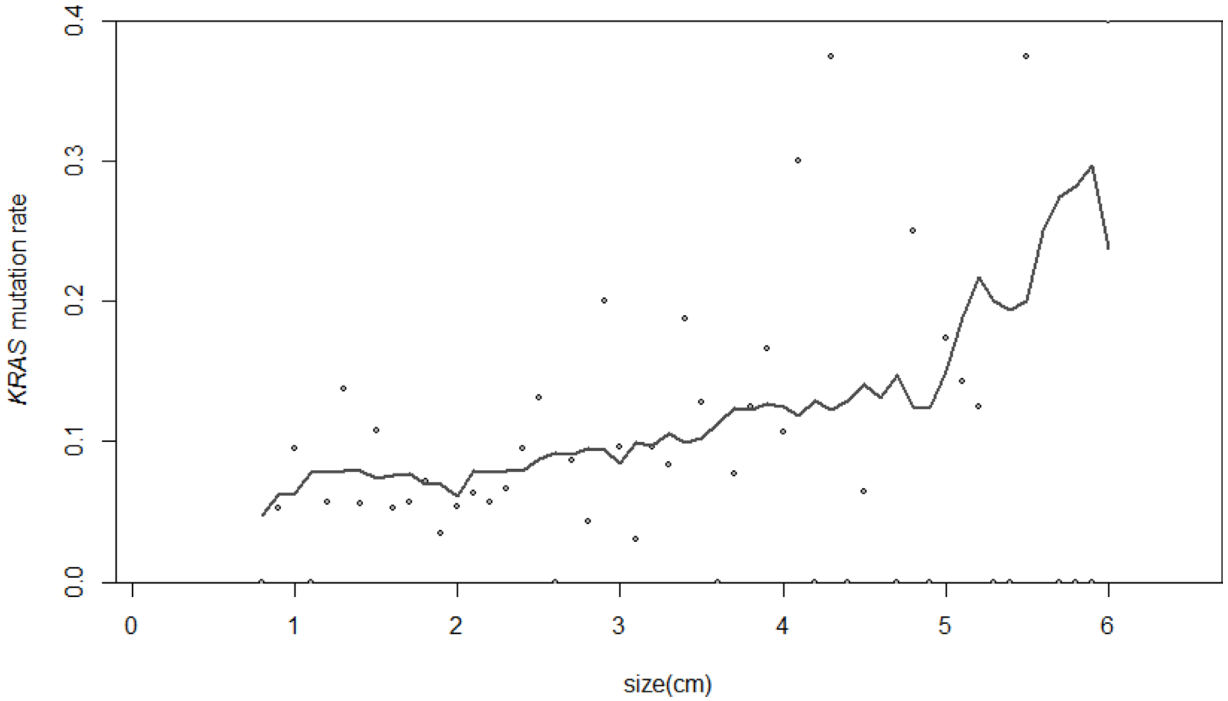

Supplementary Figure S4e: Tumor size and *KRAS* mutation rate. Dots represent *KRAS* mutations are frequent in large-sized tumor.

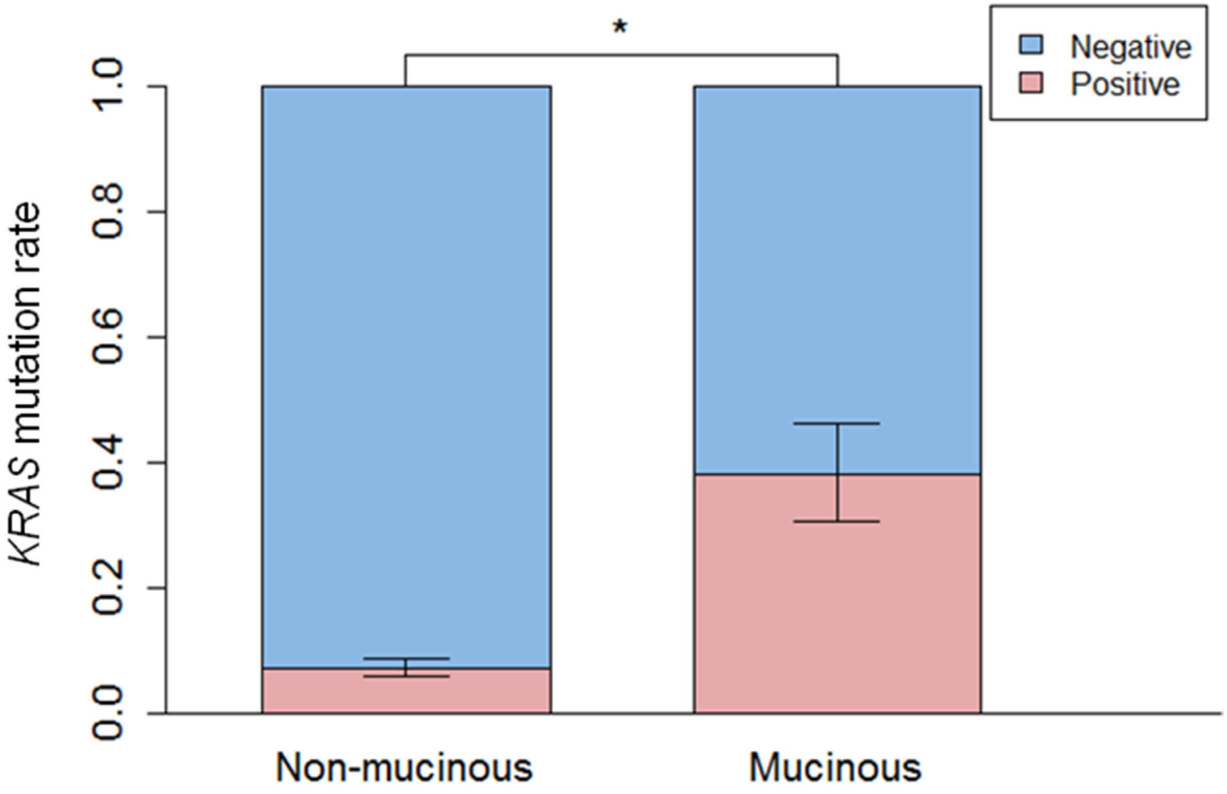

Supplementary Figure S4f: Mucinous type and *KRAS* mutation rate. *KRAS* mutations are frequent in mucinous type.

(*Continued*)

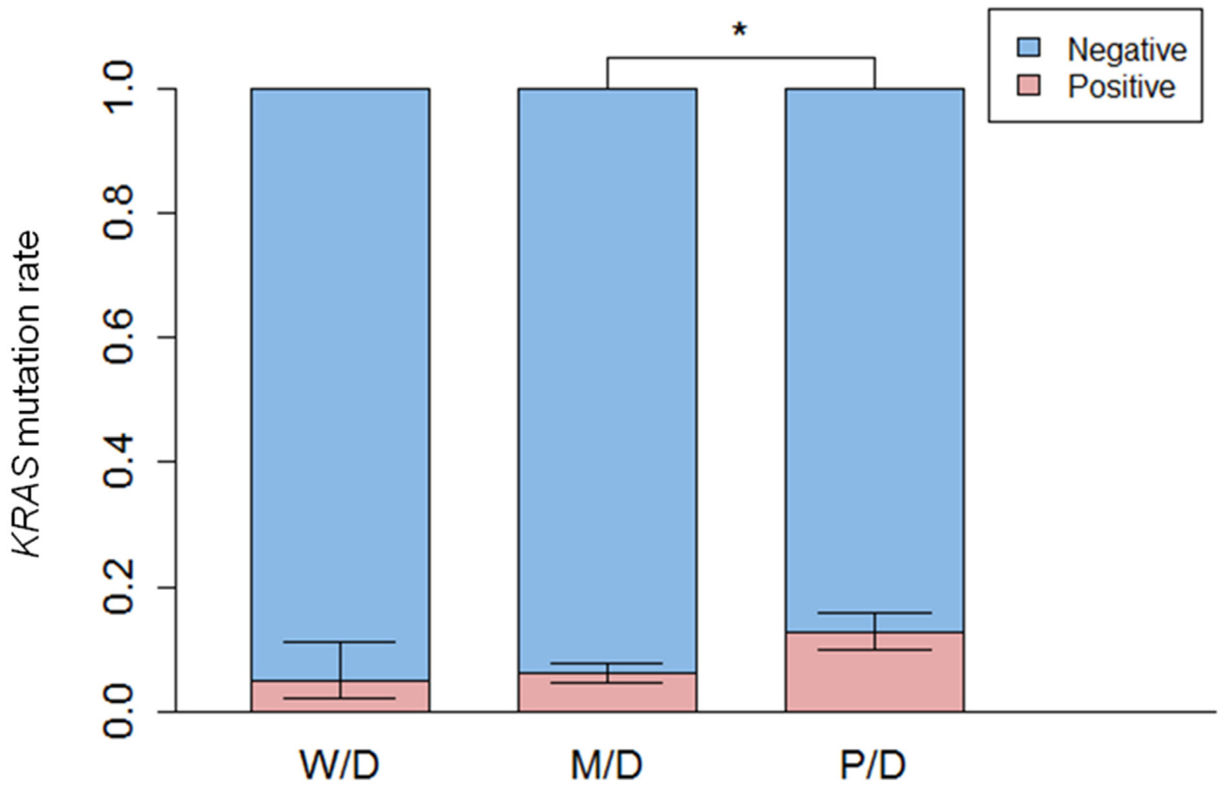

Supplementary Figure S4g: Tumor differentiation and *KRAS* mutation rate. *KRAS* mutations are frequent in poorly differentiated tumor.

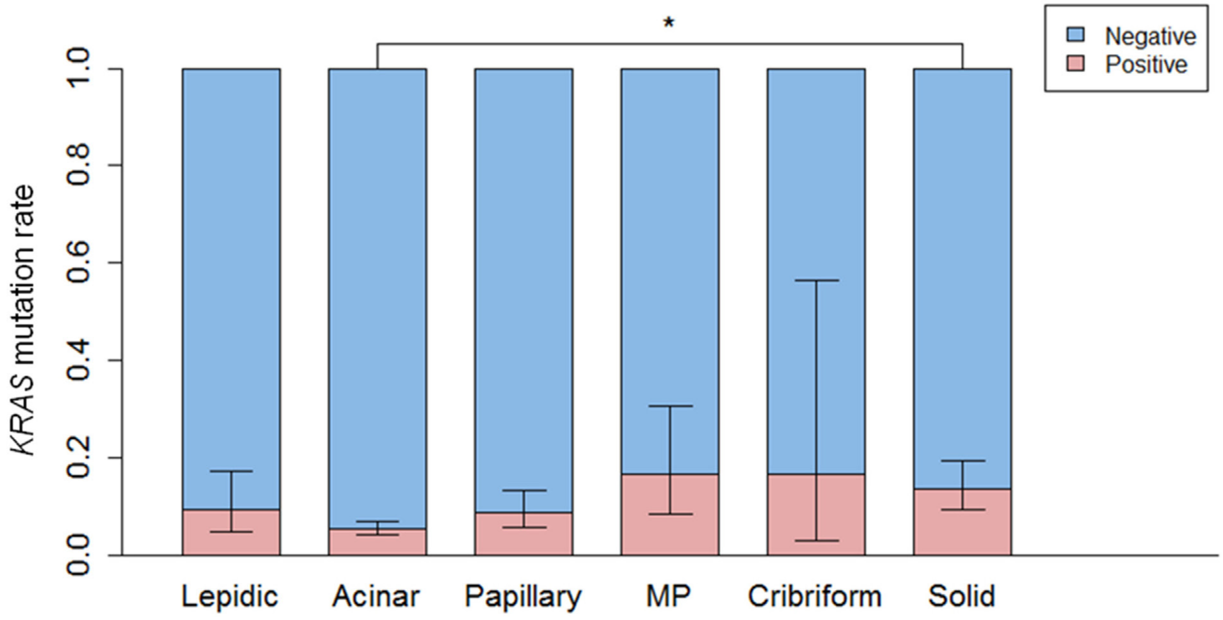

Supplementary Figure S4h: Primary histologic pattern and *KRAS* mutation rate. *KRAS* mutations are frequent in solid pattern.

(*Continued*)

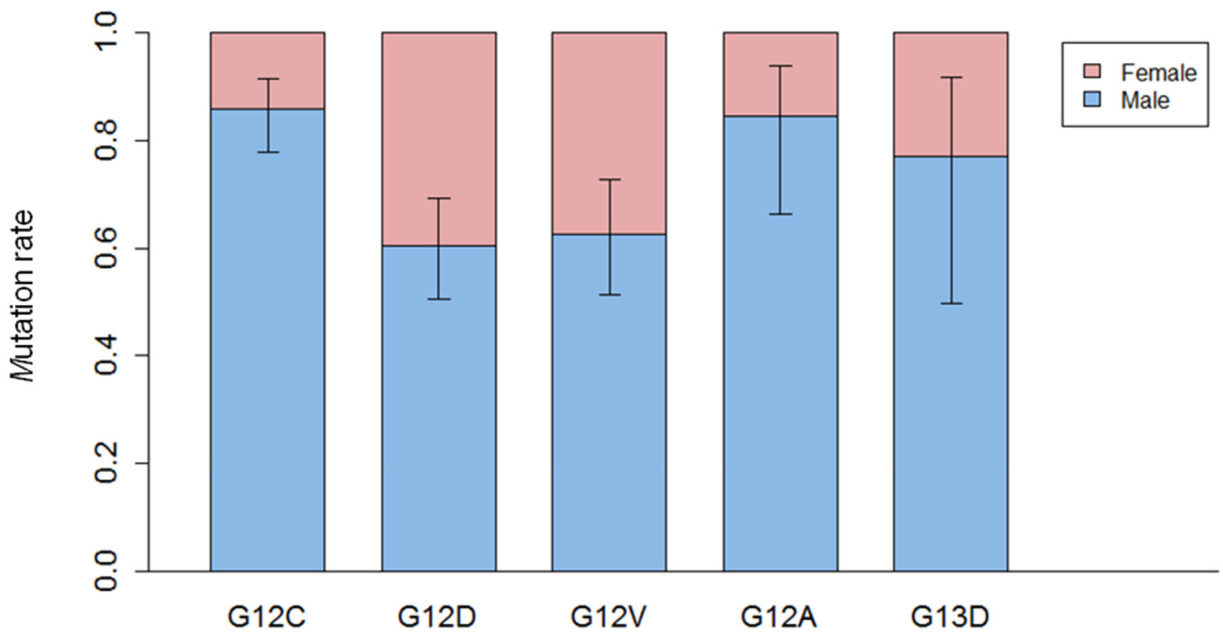

Supplementary Figure S4i: Subtypes of *KRAS* mutation and sex ratio. G12C mutations are more frequent in male patient than G12D or G12V mutations.

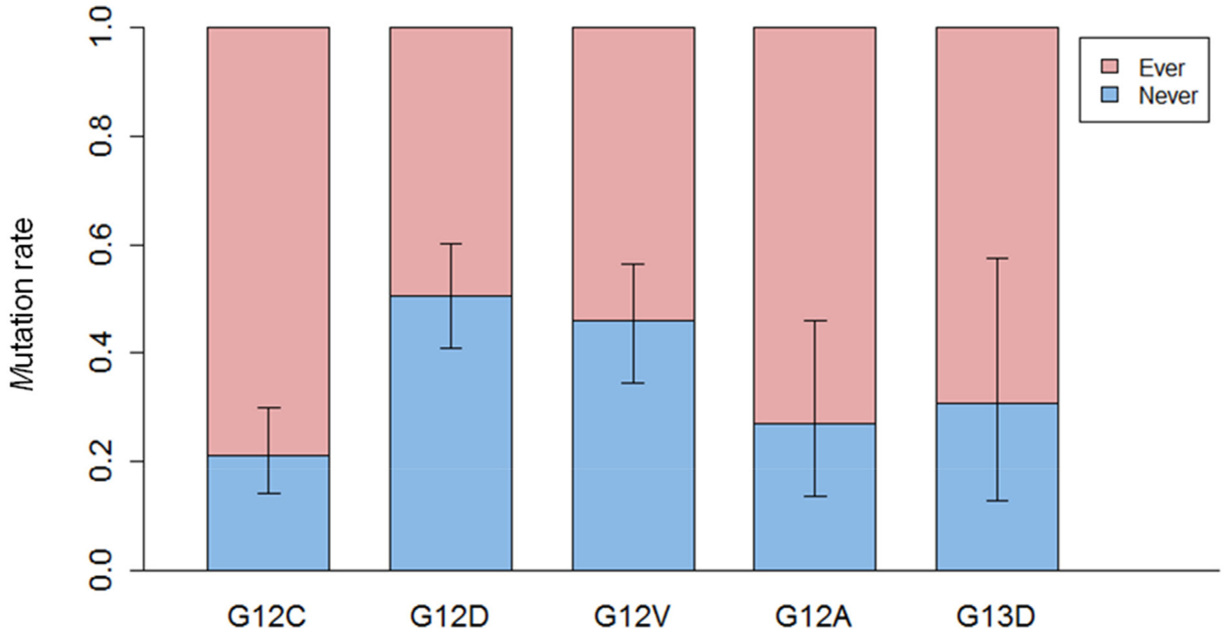

Supplementary Figure S4j: Subtypes of *KRAS* mutation and smoking ratio. G12C mutations are more frequent in smokers than G12D or G12V mutations.

Supplementary Figure S5: Association between clinicopathologic features and *ALK* rearrangement.

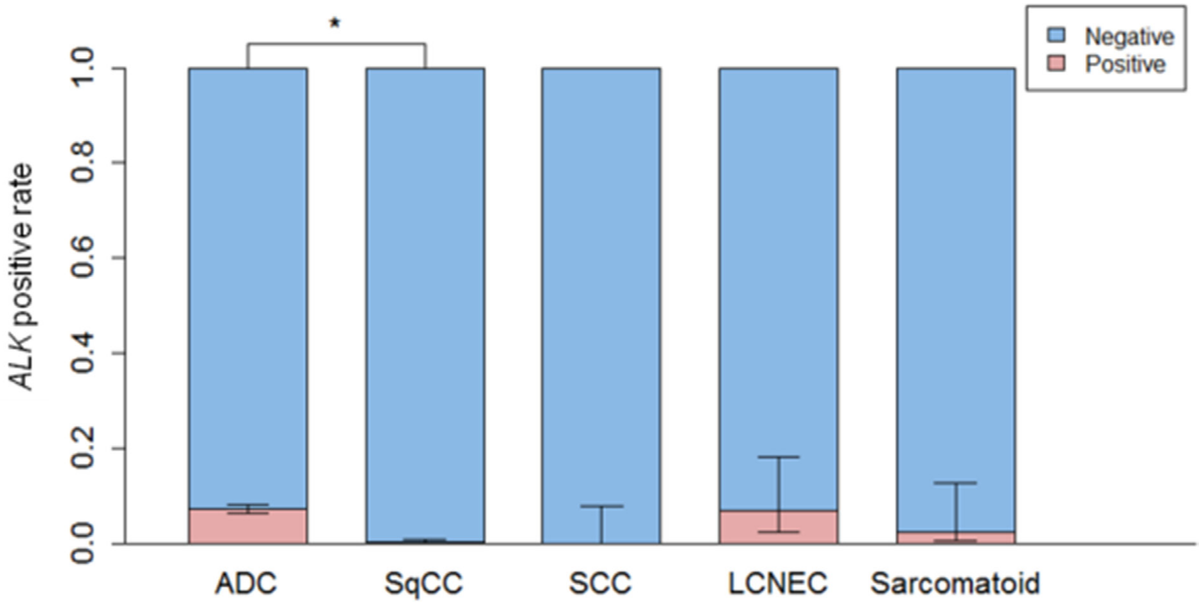

Supplementary Figure S5a: Histologic types of lung cancer and *ALK* positive rate. *ALK* rearrangements are more frequent in adenocarcinoma

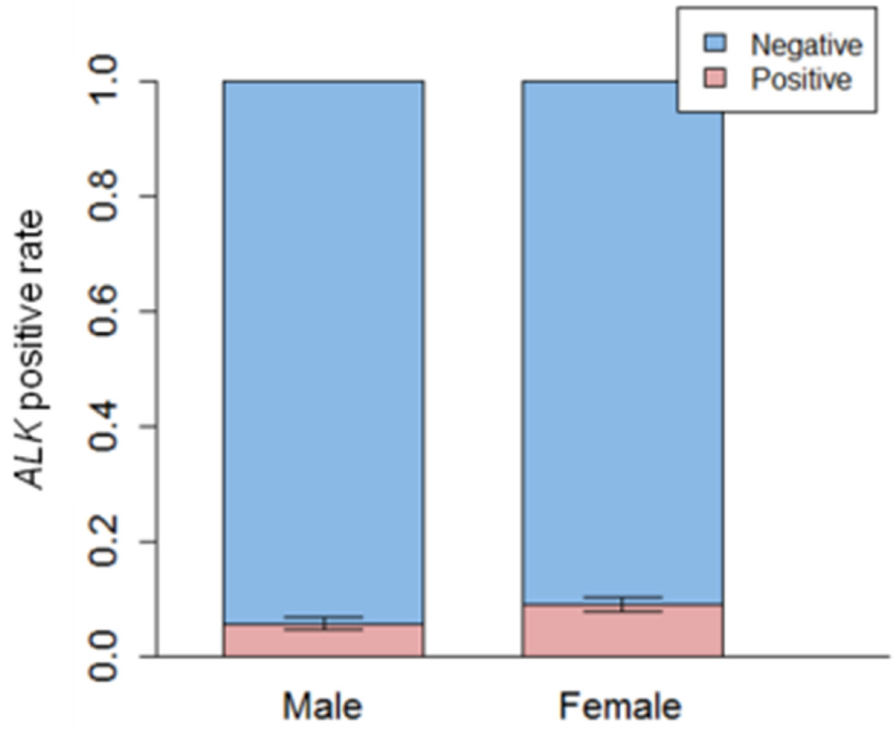

Supplementary Figure S5b: Sex and *ALK* positive rate. There is no significant difference of *ALK* rearrangement between male and female patients

(*Continued*)

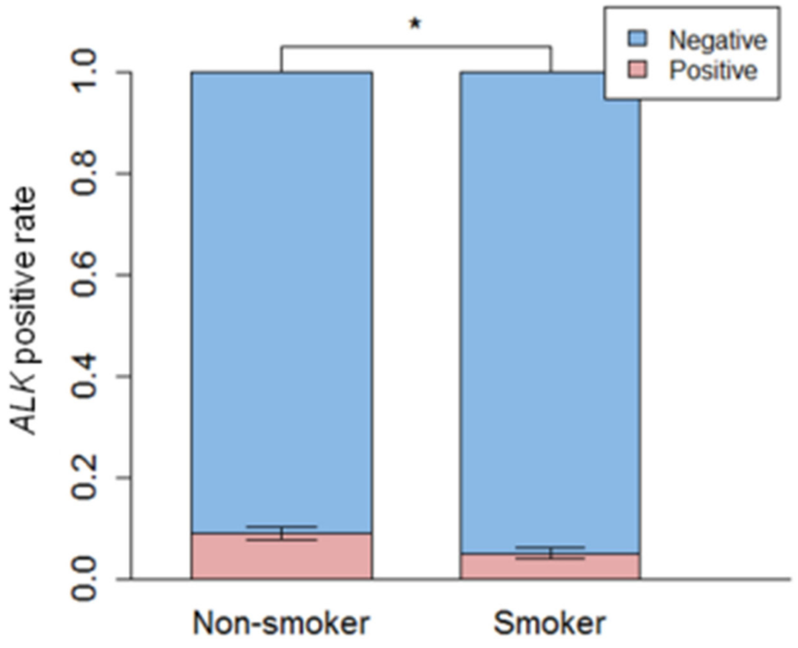

Supplementary Figure S5c: Smoking history and *ALK* positive rate. *ALK* rearrangements were more frequent in non-smoker.

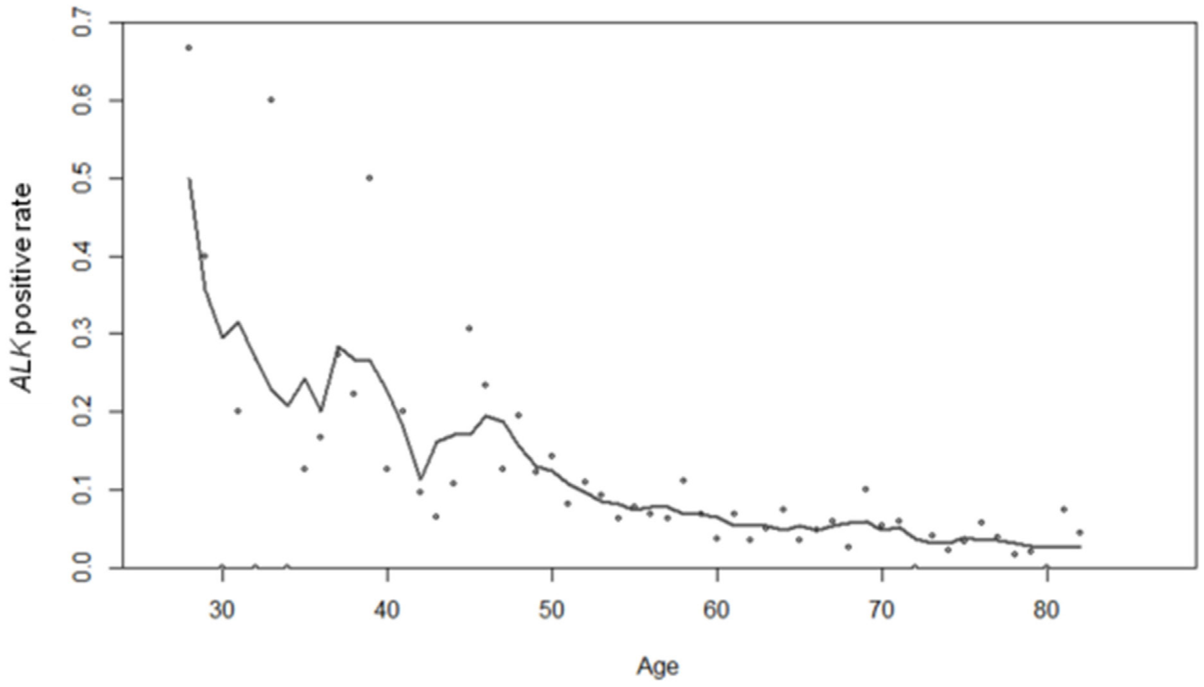

Supplementary Figure S5d: Age and *ALK* positive rate. Dots represent *ALK* rearrangement proportion at specific age. The line represents the moving average trend of *ALK* rearrangement proportion. *ALK* rearrangements are more frequent in young patients.

(*Continued*)

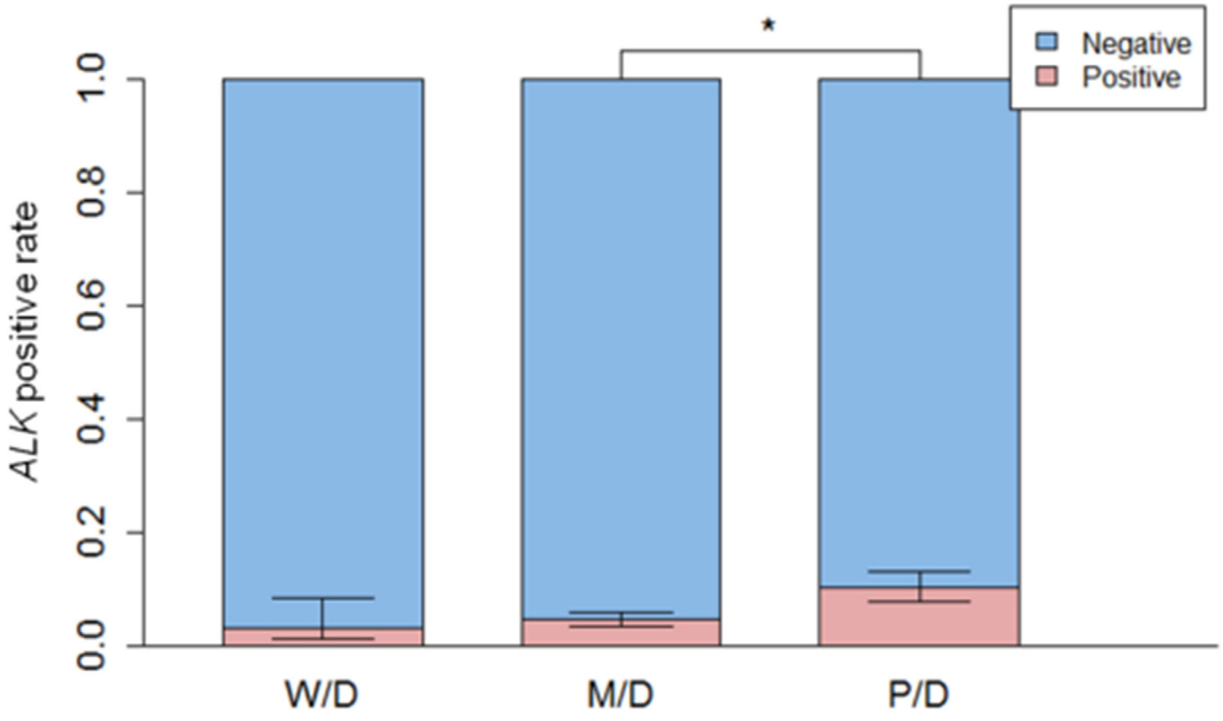

Supplementary Figure S5e: Tumor differentiation and *ALK* positive rate. *ALK* rearrangements are more frequent in poorly differentiated tumor.

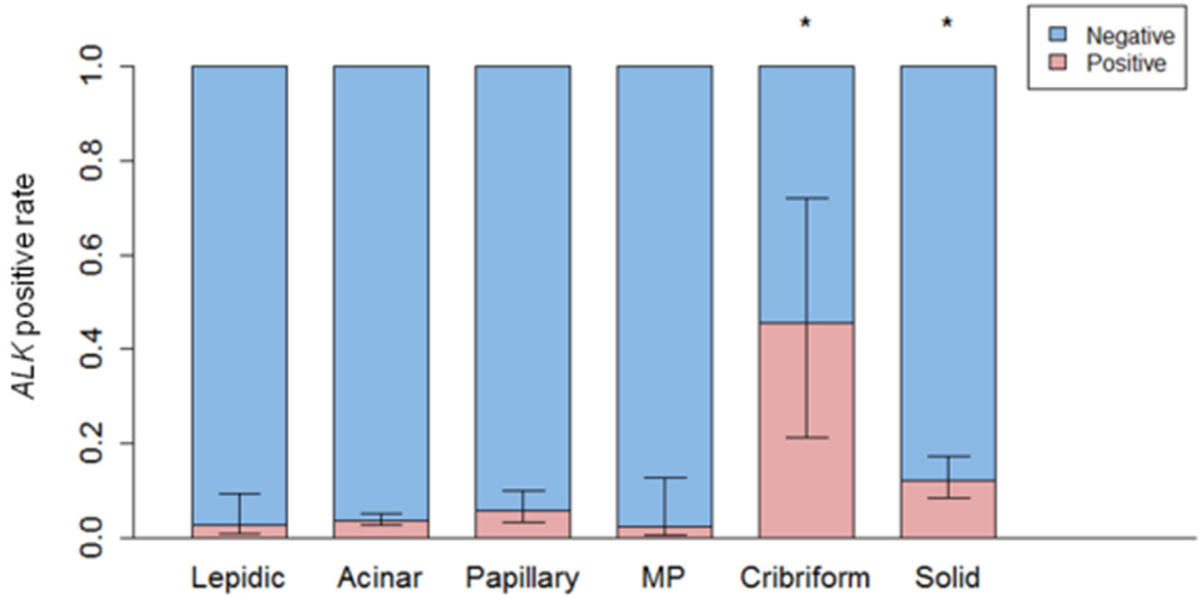

Supplementary Figure S5f: Primary pattern and *ALK* positive rate. *ALK* rearrangements are more frequent in cribriform and solid patterns.

(*Continued*)

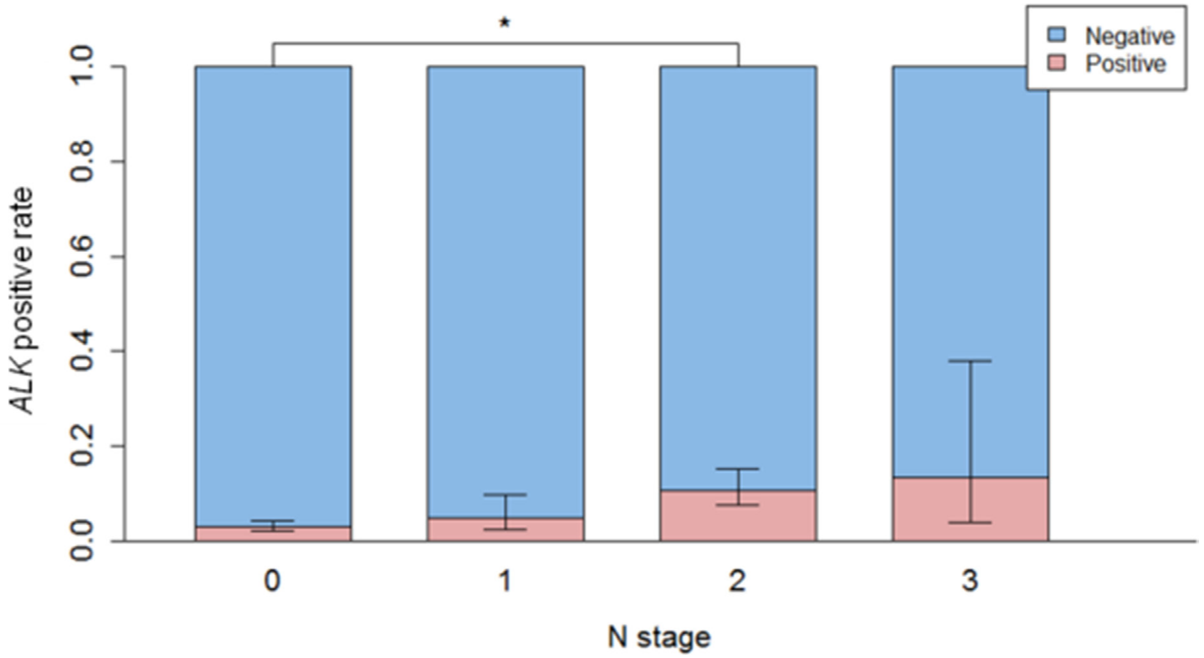

Supplementary Figure S5g: N stage and *ALK* positive rate. *ALK* rearrangements are more frequent in higher N stages.

Supplementary Figure S6: Chromatogram and histologic slide, and FISH results of tumors having both *EGFR* and *KRAS* or *EGFR* and *KRAS* mutations.

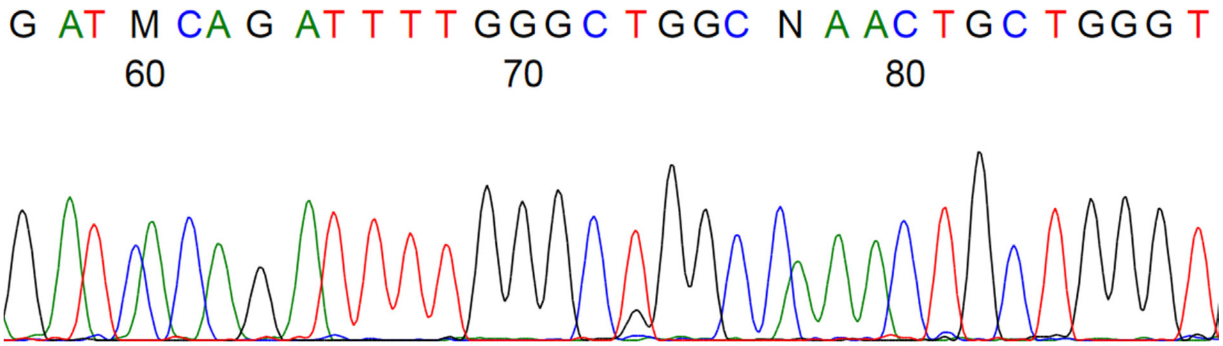

Supplementary Figure S6a: Chromatogram of *EGFR* exon 21 of DM01. L858R point mutation is detected.

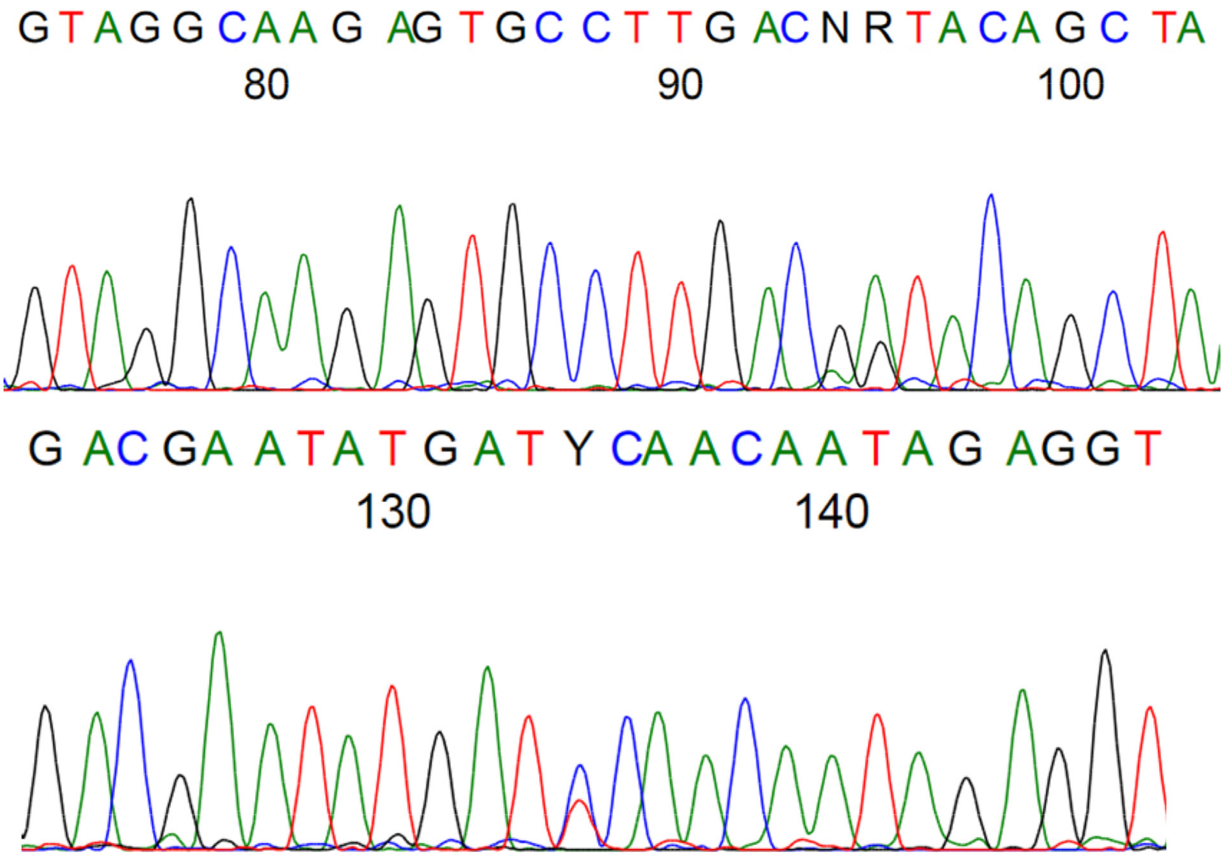

Supplementary Figure S6b: Chromatogram of *KRAS* exon 2 of DM01. I21S and P34S point mutations are detected.

(*Continued*)

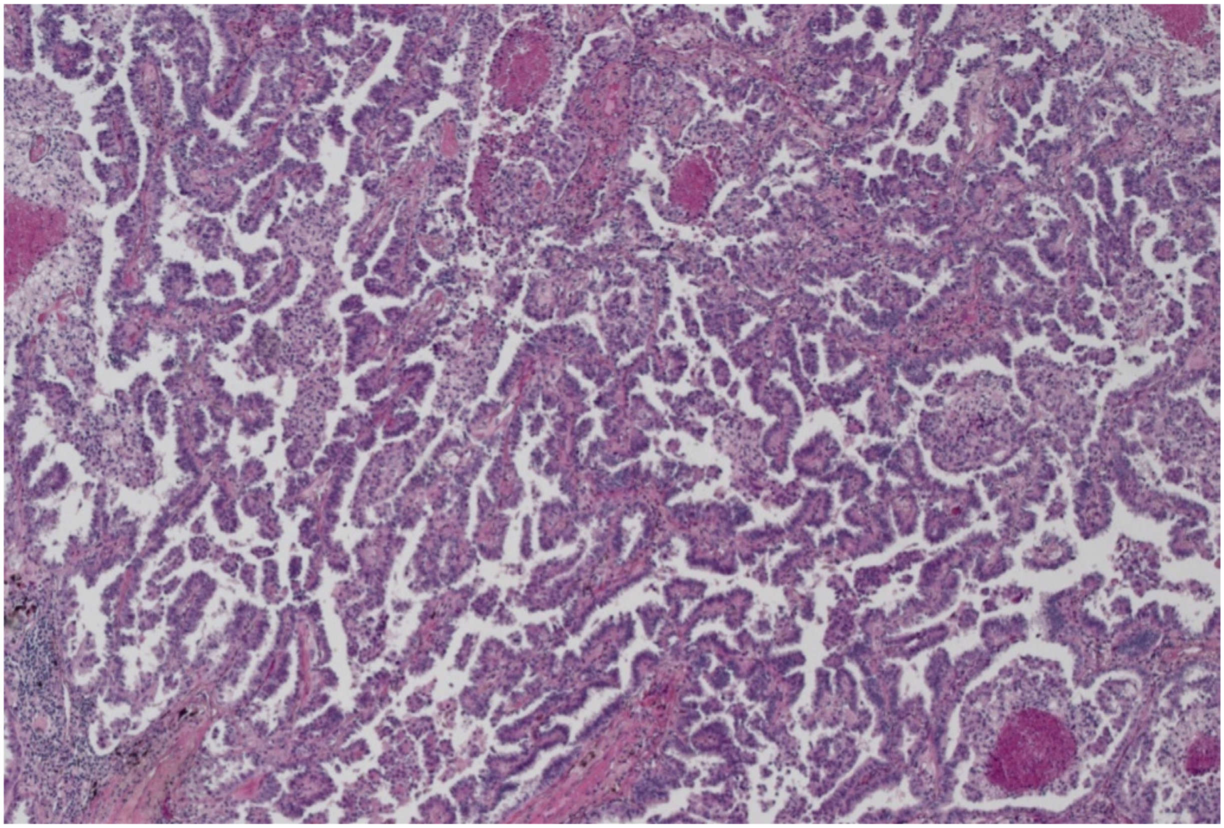

Supplementary Figure S6c: H&E slide of DM01. The tumor has primarily papillary pattern.

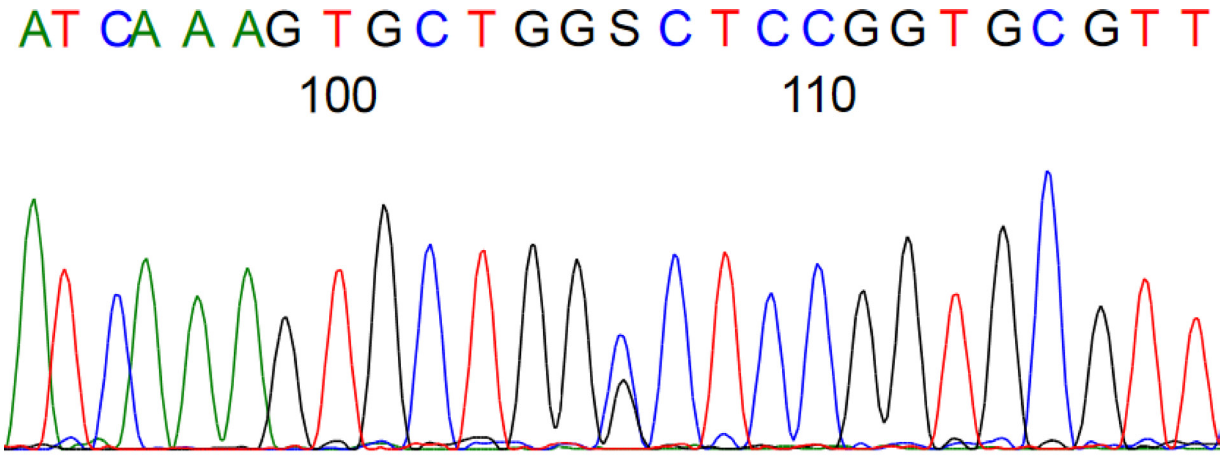

Supplementary Figure S6d: *EGFR* exon 18 chromatogram of DM02. A G719A point mutation is detected.

(*Continued*)

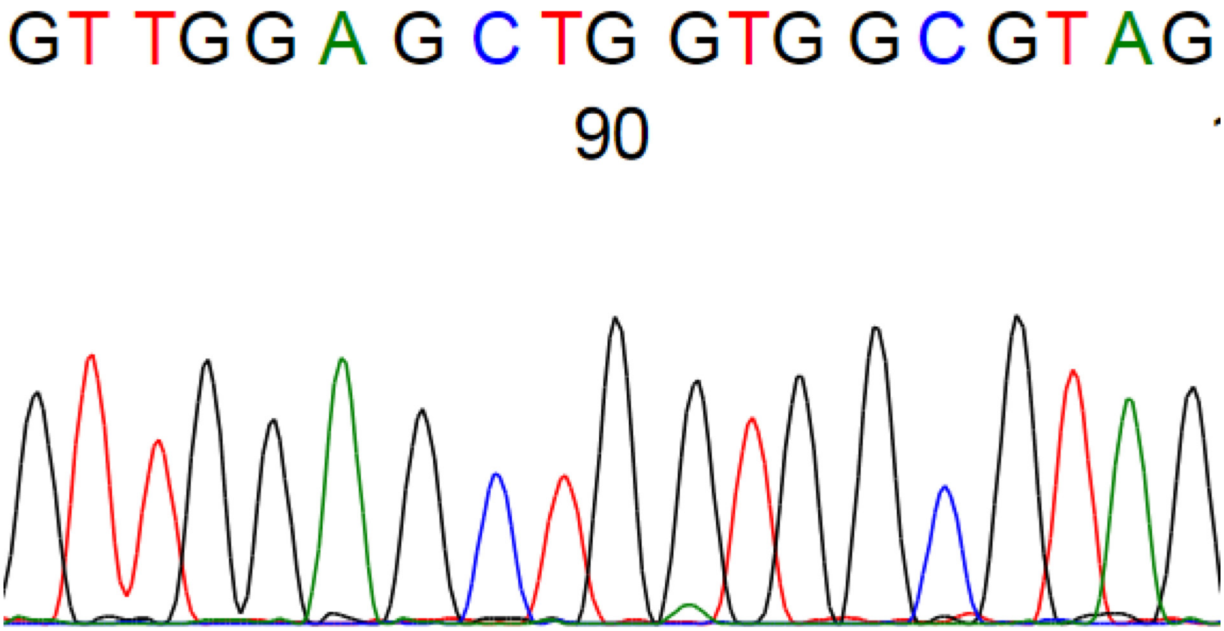

Supplementary Figure S6e: *KRAS* exon 2 chromatogram of DM02. A G12D point mutation is detected.

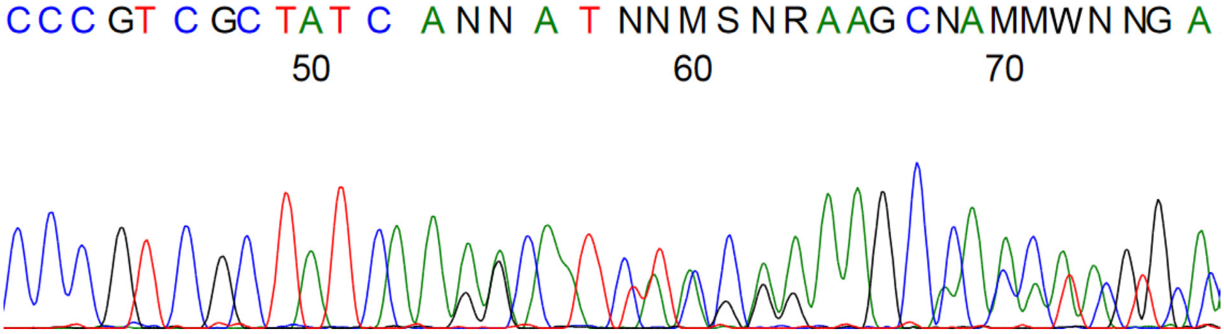

Supplementary Figure S6f: Chromatogram of *EGFR* exon 19 of DM03. 15 base pair deletion is detected.

(*Continued*)

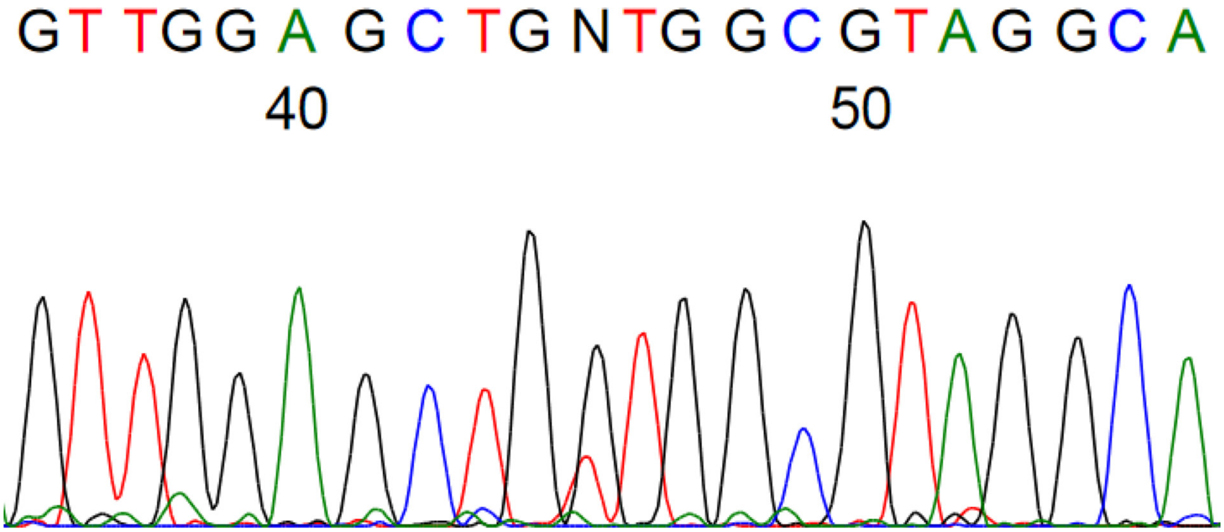

Supplementary Figure S6g: Chromatogram of *KRAS* exon 2 of DM03. G12V point mutation is identified.

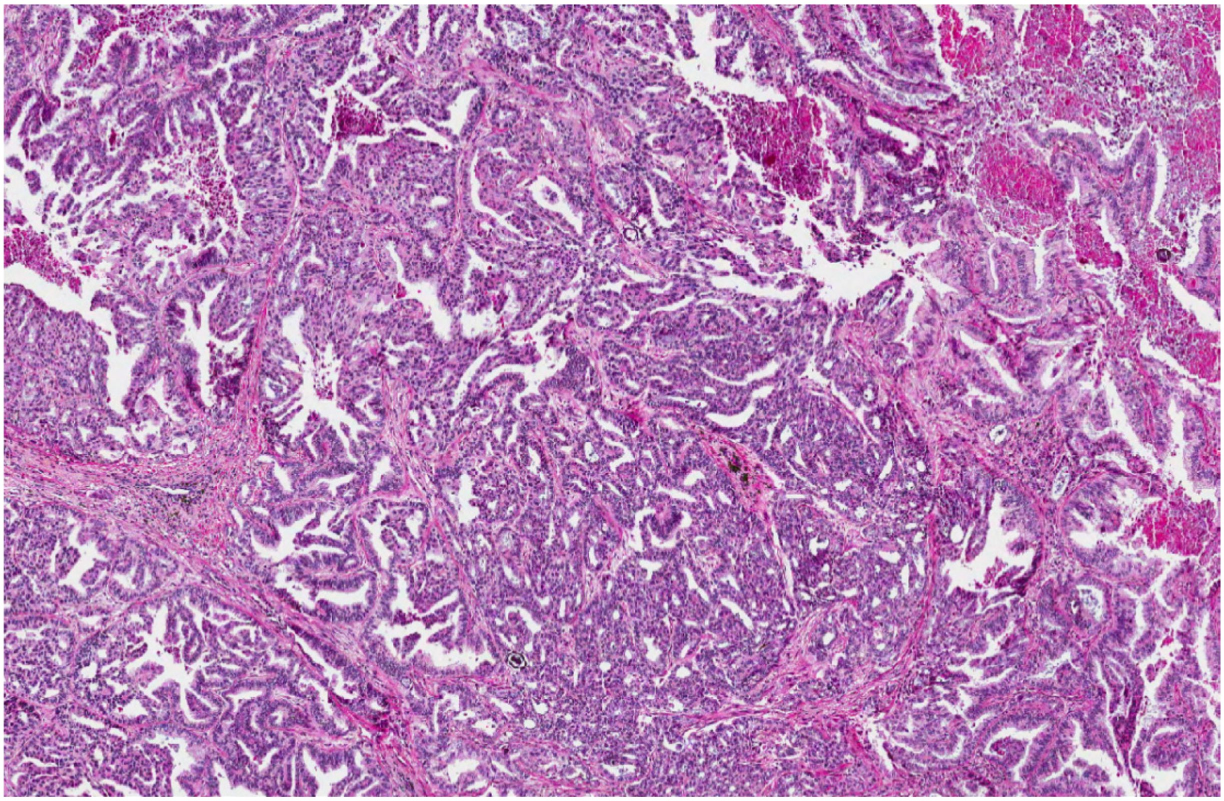

Supplementary Figure S6h: H&E slide of DM03. The tumor has primarily papillary pattern.

(Continued)

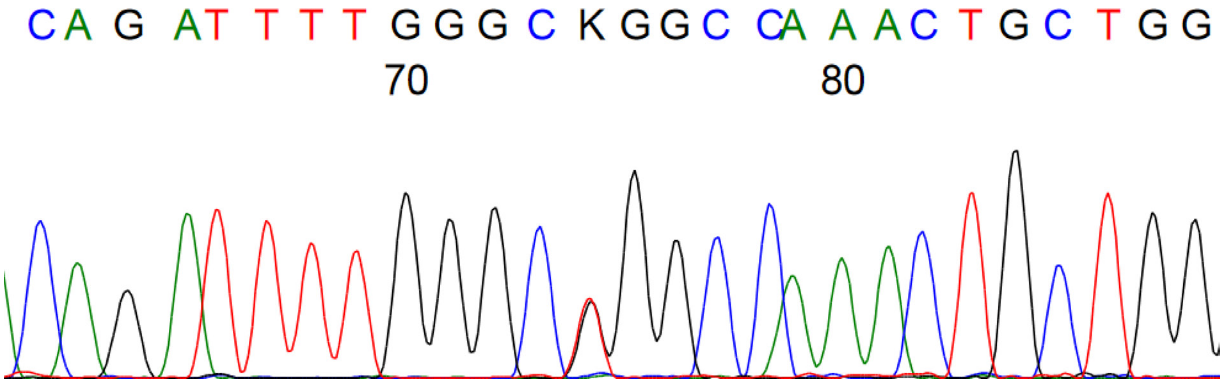

Supplementary Figure S6i: Chromatogram of *EGFR* exon 21 of DM04. L858R point mutation is detected.

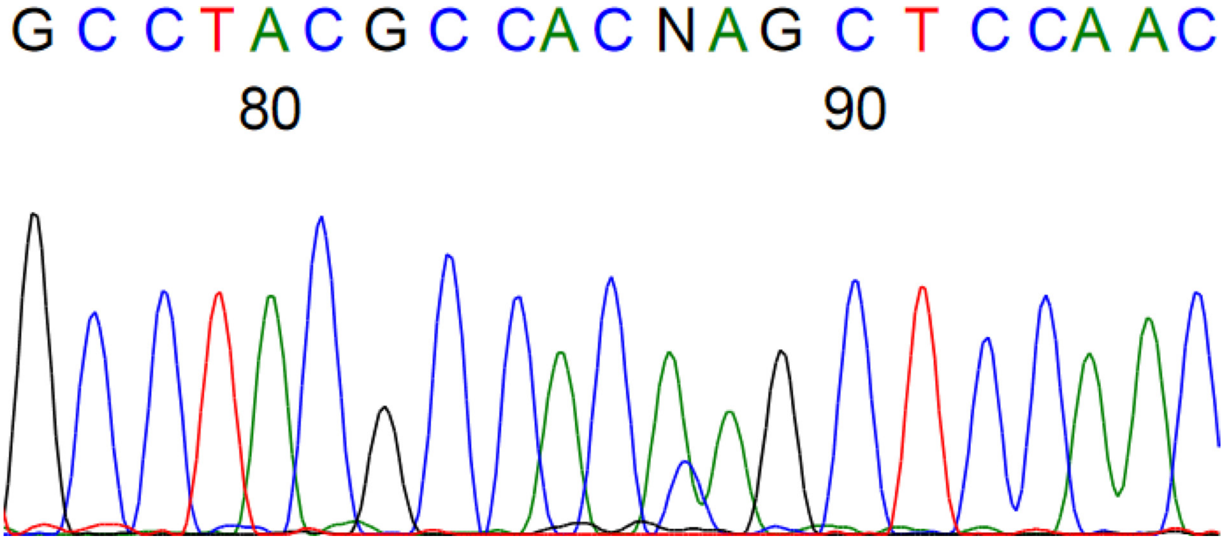

Supplementary Figure S6j: Chromatogram of *KRAS* exon 2 of DM04. G12D point mutation is detected.

(*Continued*)

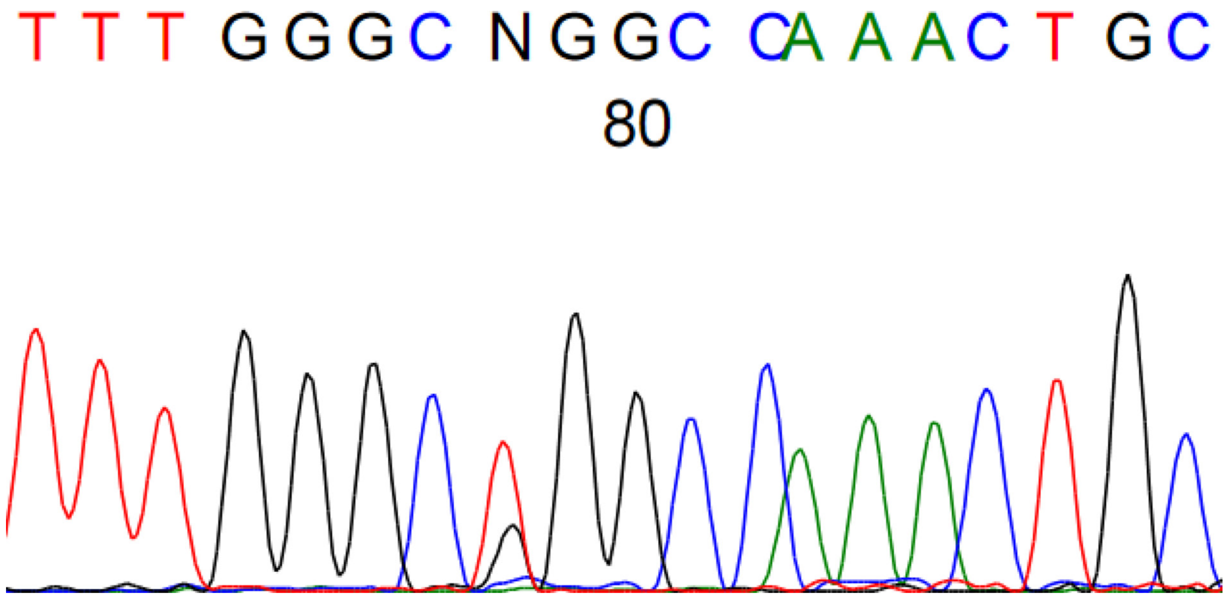

Supplementary Figure S6k: Chromatogram of *EGFR* exon 21 of DM05. L858R point mutation is detected.

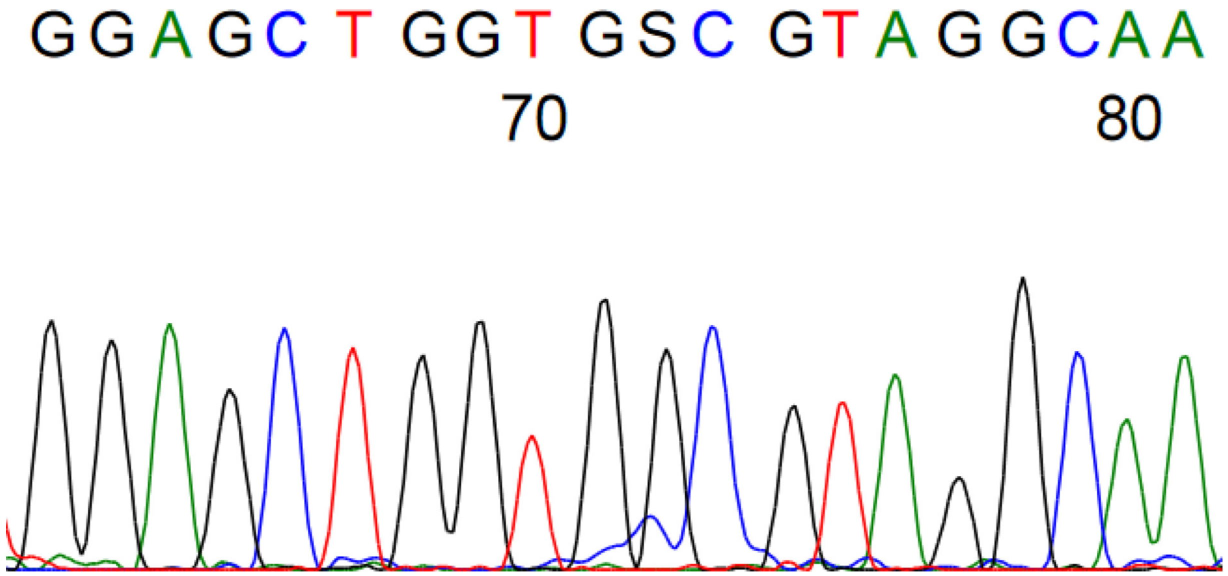

Supplementary Figure S6l: Chromatogram of *KRAS* exon 2 of DM05. G13A point mutation is detected.

(*Continued*)

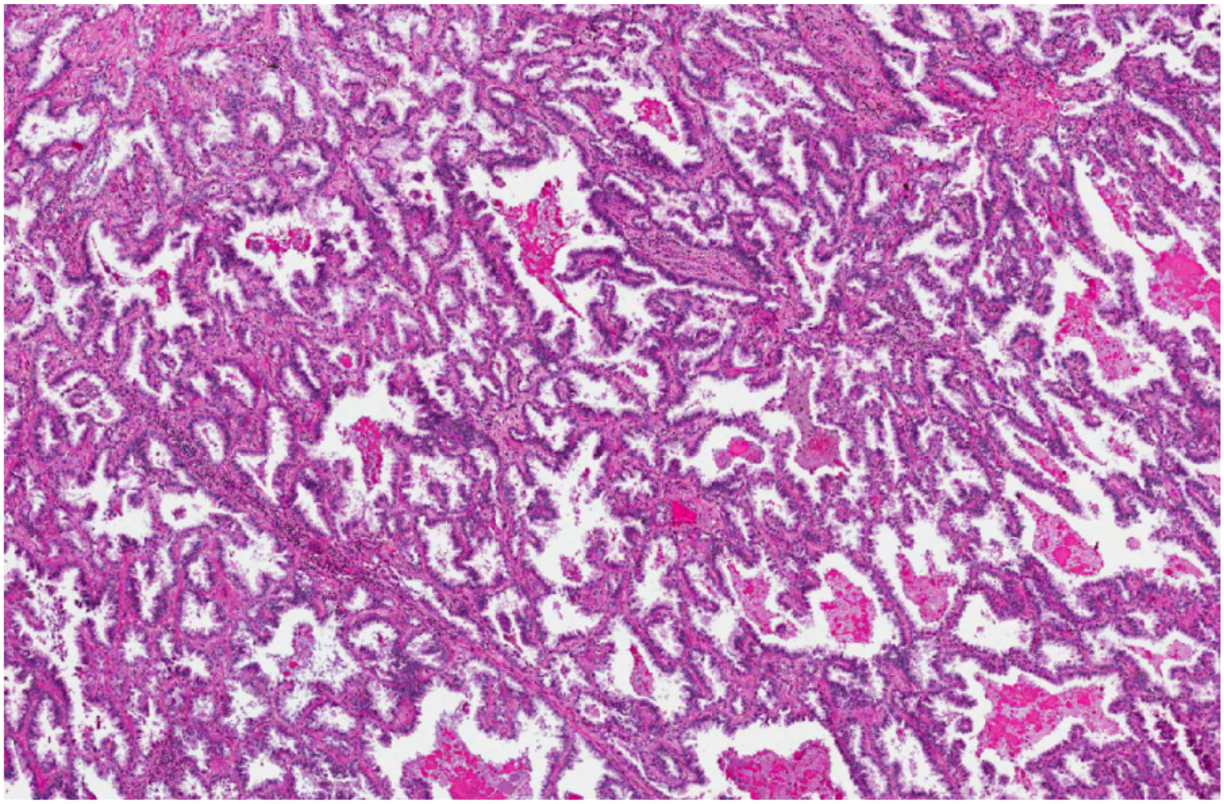

Supplementary Figure S6m: H&E slide of DM05. The tumor has primarily papillary pattern.

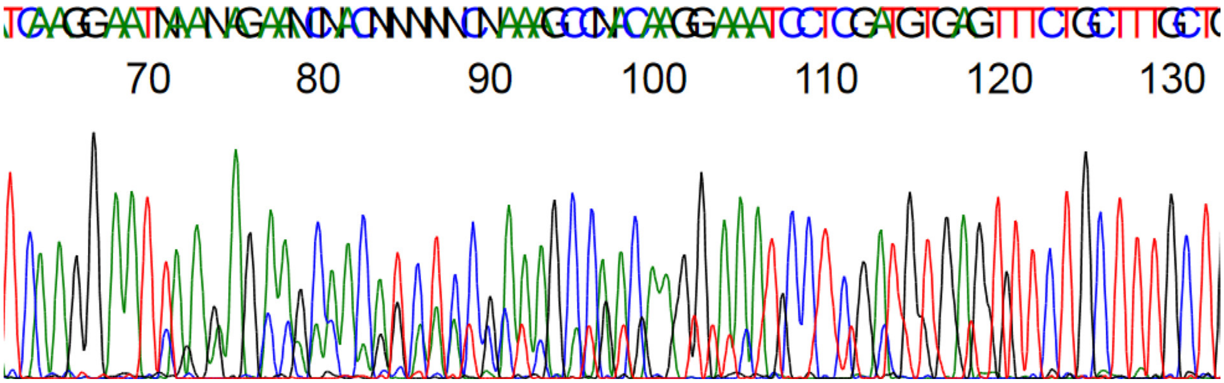

Supplementary Figure S6n: *EGFR* exon 19 chromatogram of DM06. 18 base-pairs are deleted.

(Continued)

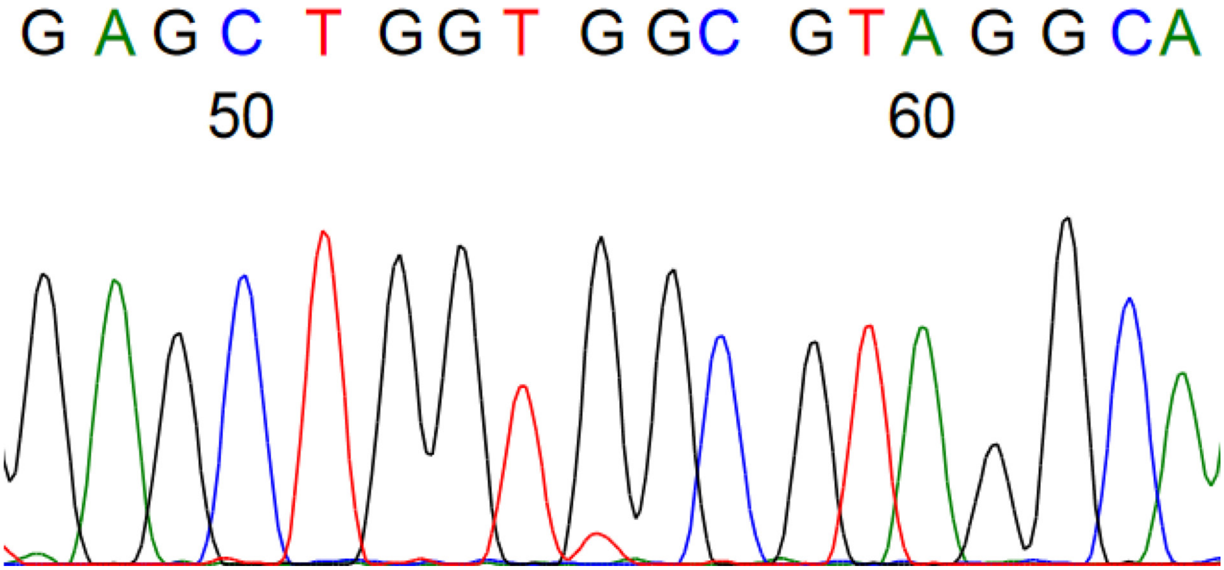

Supplementary Figure S6o: *KRAS* exon 2 chromatogram of DM06. A G13C point mutation is detected.

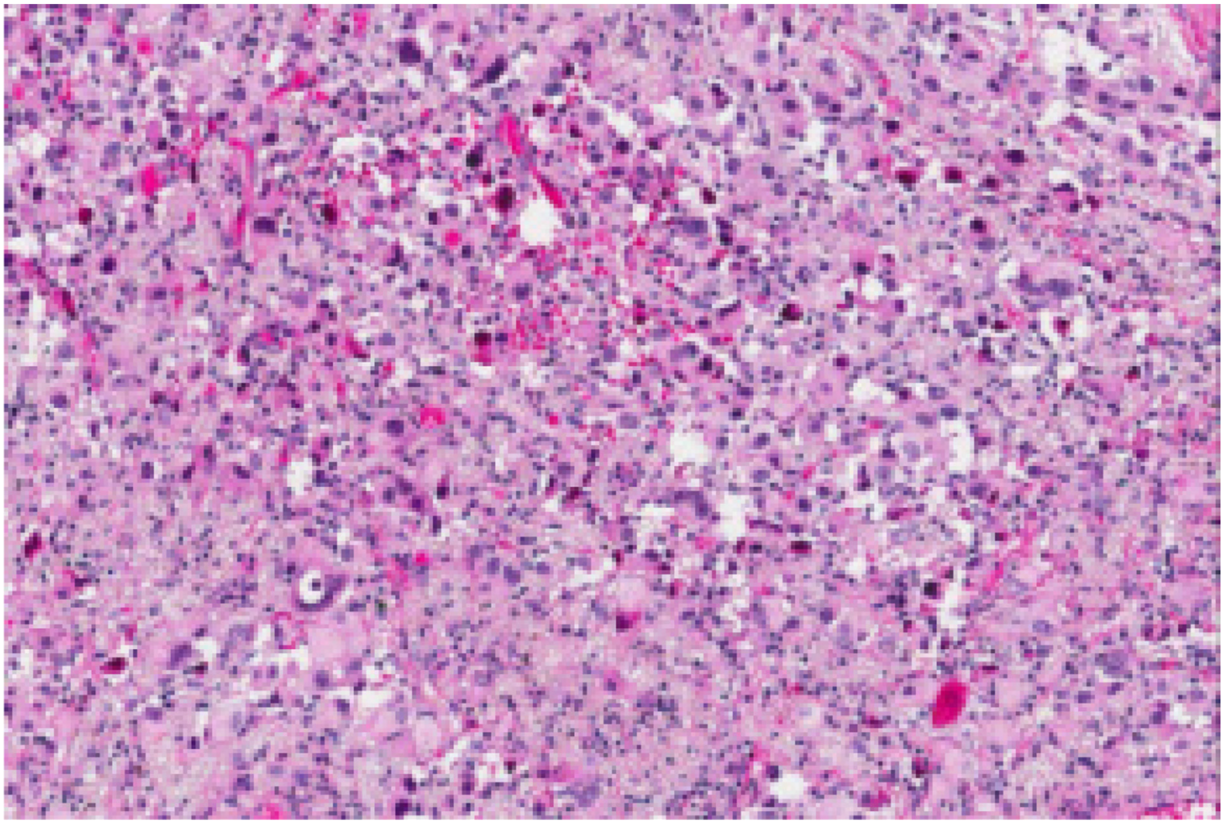

Supplementary Figure S6p: H&E slide of DM06. The tumor has primarily solid pattern.

(*Continued*)

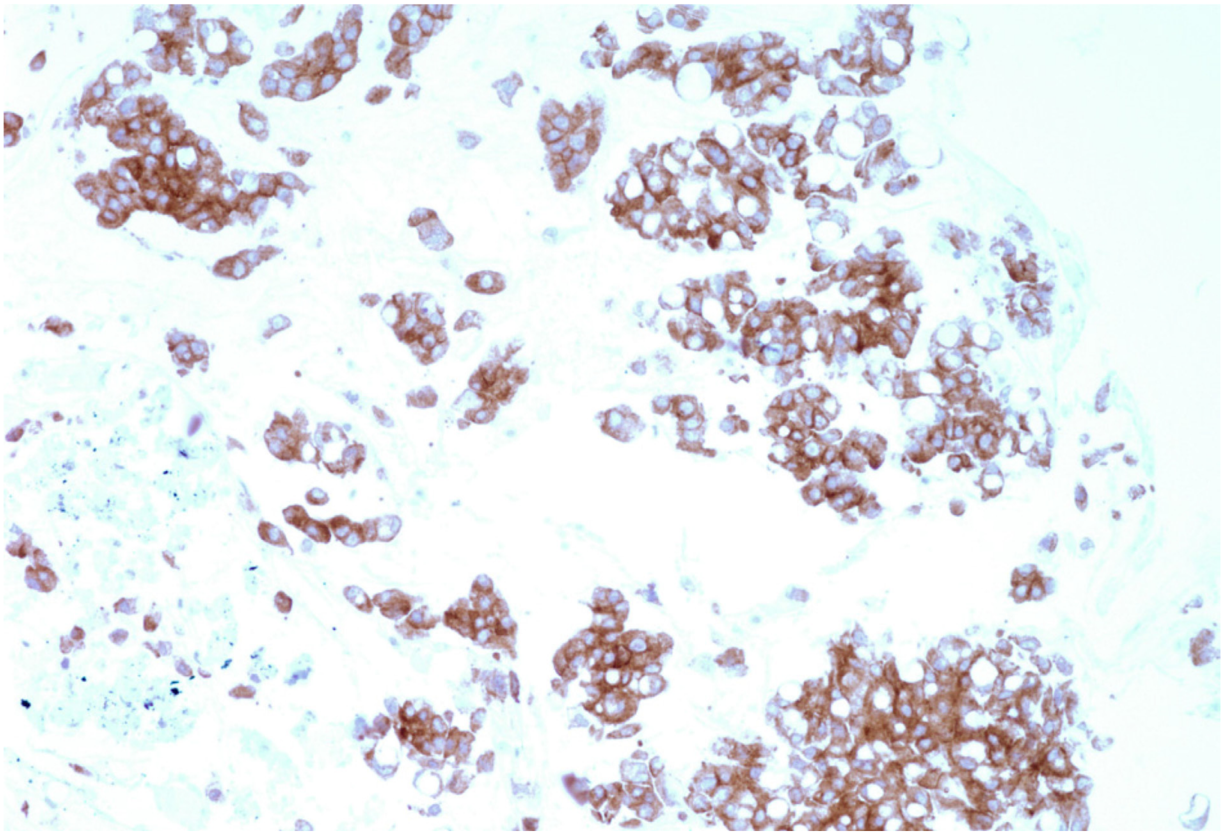

Supplementary Figure S6q: Immunohistochemistry of ALK of DM07. Diffuse cytoplasmic stain is observed.

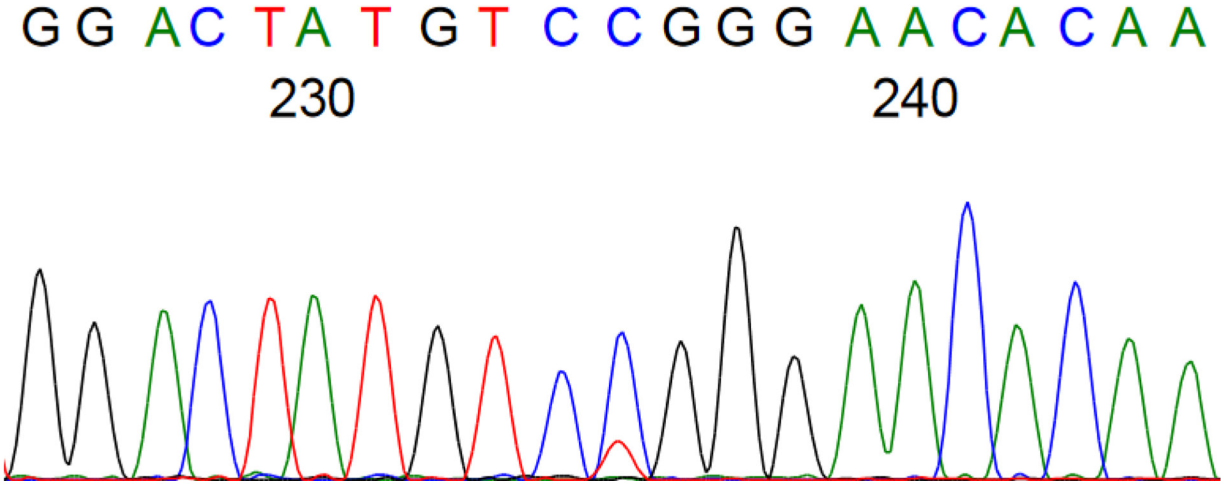

Supplementary Figure S6r: *EGFR* exon 20 chromatogram of DM08. A R803W point mutation is identified.

(*Continued*)

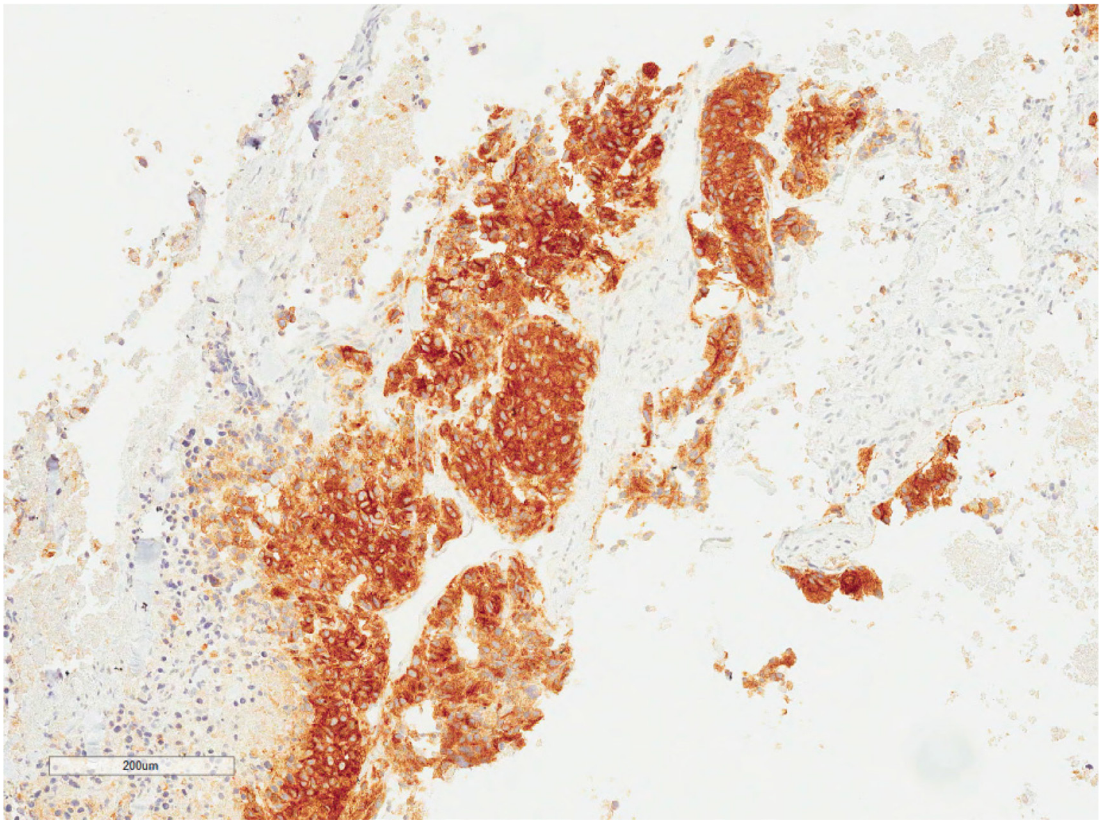

Supplementary Figure S6s: *ALK* immunohistochemistry staining of DM08. Diffuse cytoplasmic staining is observed.

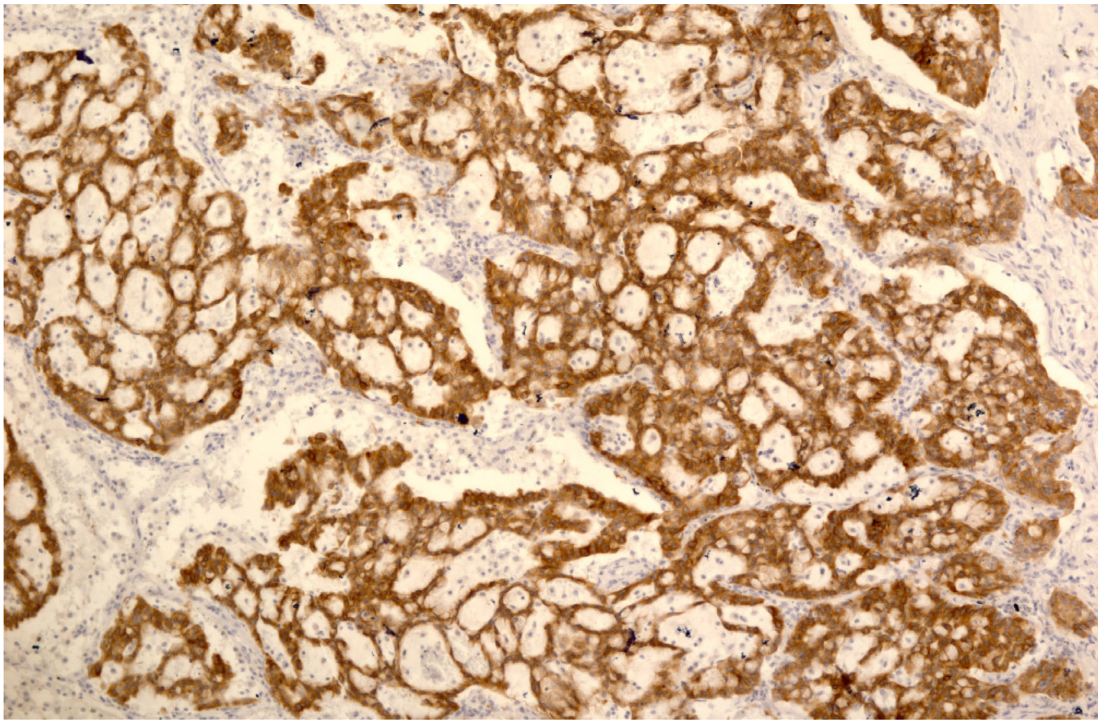

Supplementary Figure S6t: Immunohistochemistry of ALK of DM09. Diffuse cytoplasmic stain is observed.

(*Continued*)

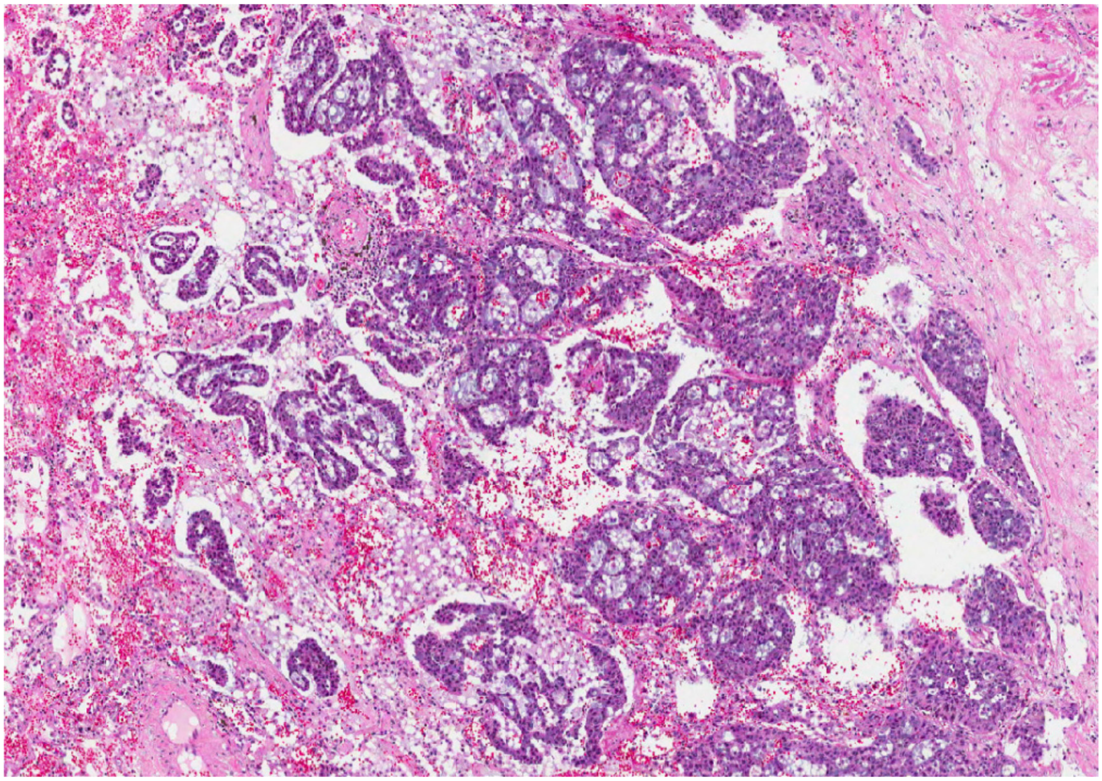

Supplementary Figure S6u: H&E slide of DM09. The tumor has cribriform pattern.

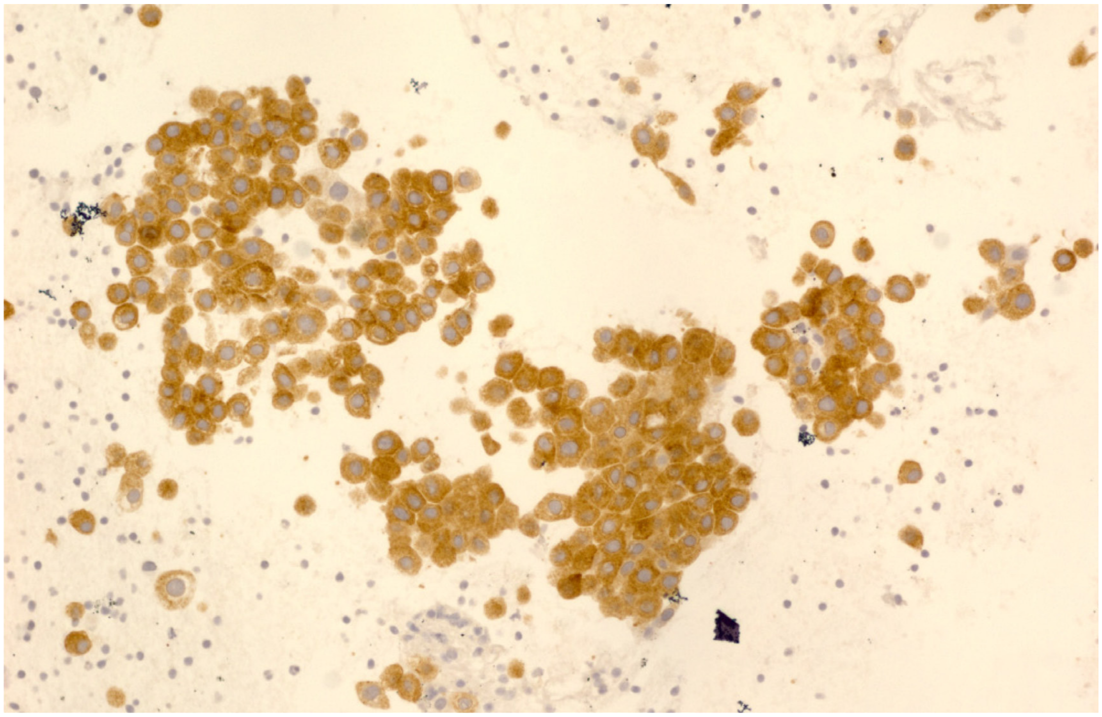

Supplementary Figure S6v: Immunohistochemistry of ALK of DM10. Diffuse cytoplasmic stain is observed.

(*Continued*)

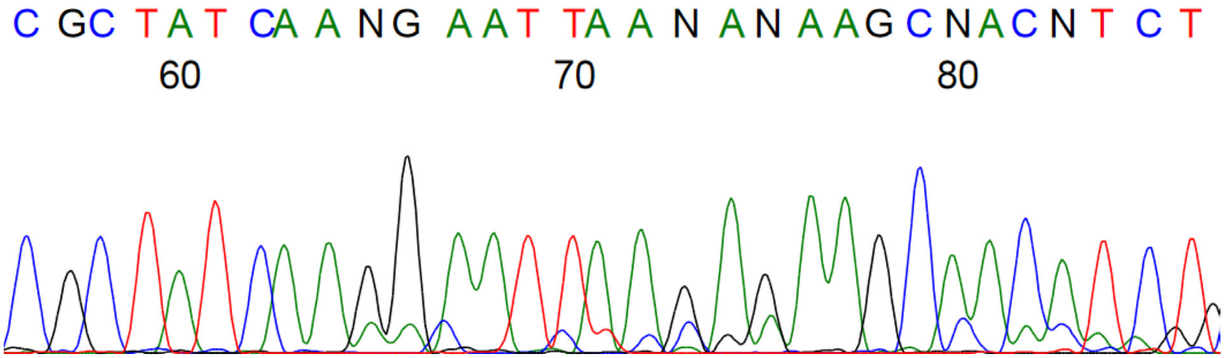

Supplementary Figure S6w: Chromatogram of *EGFR* exon 19 of DM11. 15 base pair deletion is detected.

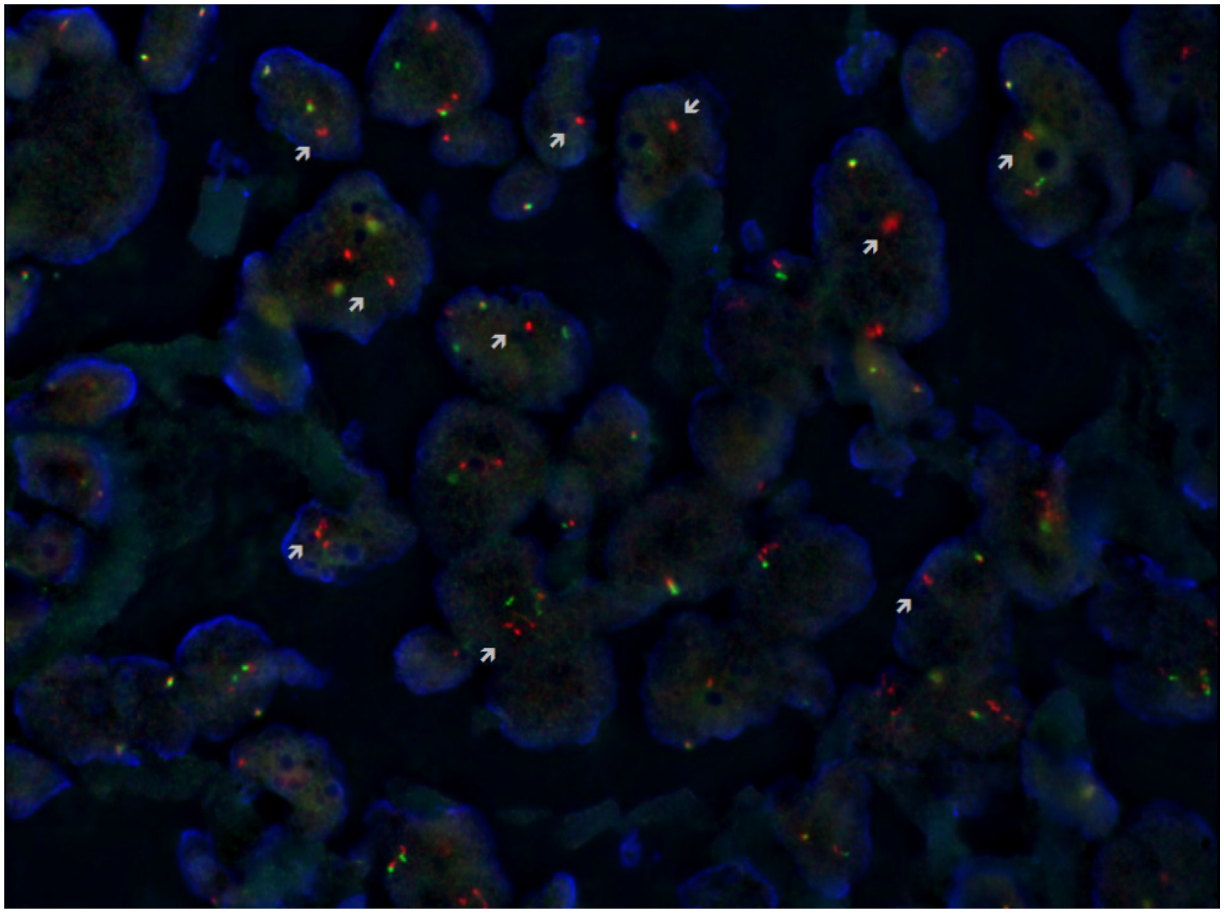

Supplementary Figure S6x: *ALK* FISH of DM11. Separate probe signals are detected.

(*Continued*)

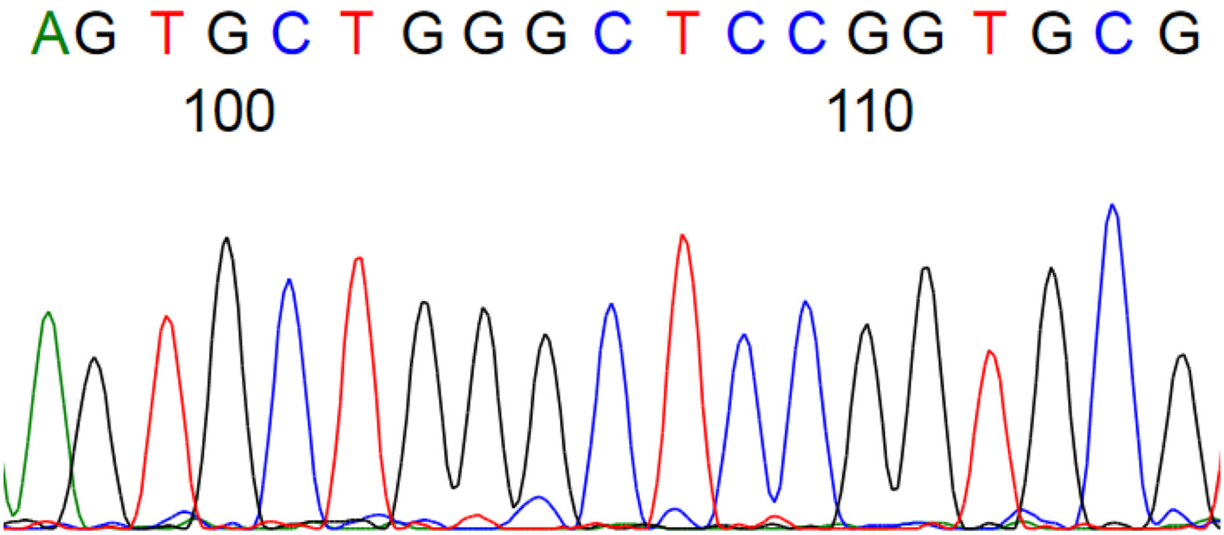

Supplementary Figure S6y: Chromatogram of *EGFR* exon 18 of DM12. G719A point mutation is detected.

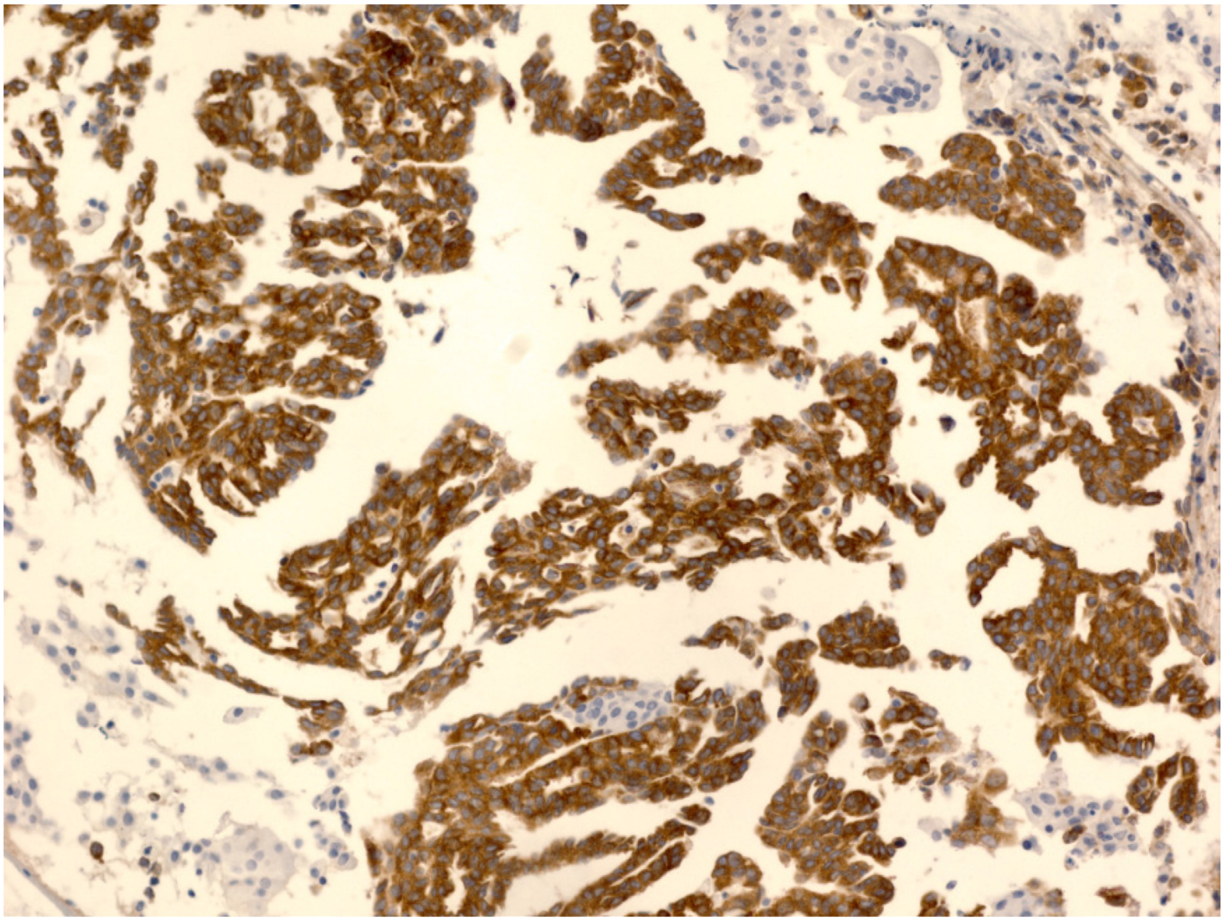

Supplementary Figure S6z: Immunohistochemistry of ALK of DM12. Diffuse cytoplasmic stain is observed.

(*Continued*)

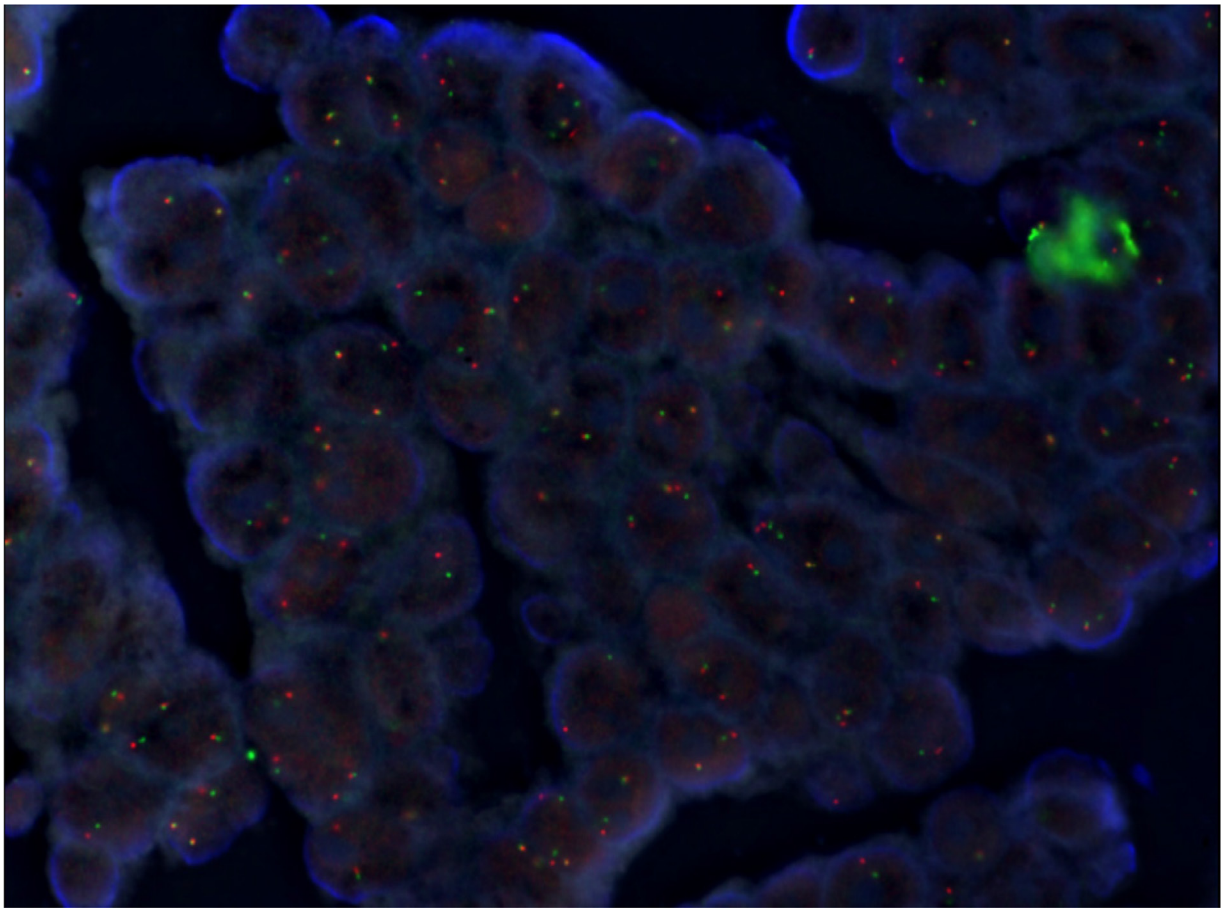

Supplementary Figure S6aa: ***ALK*** **FISH of DM12.** Separate probe signals are detected.

Supplementary Table S1: Summary of test samples

Procedure	Biopsy	4,322 (61.9)
	Resection	2,548 (36.5)
	Cytology	115 (1.6)
Bx site	Lung	4,407 (62.8)
	LN	988 (14.1)
	Bronchus	1,059 (15.1)
	Pleura	267 (3.8)
	Bone	108 (1.5)
	Body fluid	66 (0.9)
	Brain	67 (1)
	Liver	56 (0.8)
Test methods	Both PNA-clamping and Sanger sequencing	3,534 (49)
	PNA-clamping	810 (11.2)
	Sanger sequencing	2,861 (39.7)
Tumor proportion (%)	0-19	298 (690)
	20-100	1985 (3262)

Supplementary Table S2: Association between test variables and *EGFR* mutation results

		Univariate analysis			Multivariate analysis		
		**OR**	**95% CI**	***P*-value**	**OR**	**95% CI**	***P*-value**
Procedure							
vs. resection	Biopsy	0.78	0.71 - 0.87	<0.001	1.17	1.03 - 1.34	0.020
	Cytology	1.50	1.03 - 2.19	0.032	0.83	0.18 - 3.06	0.786
Bx site							
vs. lung	LN	0.53	0.46 - 0.62	<0.001	0.57	0.46 - 0.69	<0.001
	Bronchus	0.37	0.31 - 0.43	<0.001	0.67	0.54 - 0.84	<0.001
	Pleura	1.14	0.89 - 1.47	0.292	0.80	0.59 - 1.07	0.132
	Bone	1.09	0.74 - 1.60	0.664	1.16	0.72 - 1.87	0.550
	Body fluid	1.77	1.09 - 2.92	0.022	1.25	0.19 - 9.45	0.820
	Brain	1.16	0.71 - 1.89	0.554	0.98	0.54 - 1.79	0.951
	Liver	1.04	0.61 - 1.77	0.877	0.60	0.30 - 1.17	0.135
Test methods							
vs. both.	PNA only	1.08	0.93 - 1.27	0.315	1.10	0.92 - 1.32	0.304
	Sanger only	0.86	0.77 - 0.95	0.003	0.82	0.71 - 0.94	0.004
Tumor proportion	per 1%	1.00	1.00 - 1.00	0.136	1.01	1.00 - 1.01	<0.001
vs. 20-100	0-19	0.71	0.61 - 0.82	<0.001	0.71	0.60 - 0.84	<0.001

OR: odds ratio, CI: confidence interval, LN: lymph node, PNA: PNA-clamping method, Sanger: Sanger sequencing method

Supplementary Table S3: Summary of clinical variables

Variables		Total Tumors Number (percentage)	Adenocarcinoma Number (percentage)
Sex	Male	3,917 (59)	2,545 (50.7)
	Female	2,720 (41)	2,470 (49.3)
Age (years)	Mean ± SD	63.3 ± 11	62.3 ± 10.9
	40-64	3,086 (46.5)	2,569 (51.2)
	≥ 65	3,411 (51.4)	2,336 (46.6)
	< 40	140 (2.1)	110 (2.2)
Smoking	Never	3,190 (49)	2,859 (58.2)
	Ever	3,316 (51)	2,051 (41.8)
Tumor type	ADC	5,015 (75.6)	
	SqCC	1,155 (17.4)	
	SCC	67 (1)	
	LCNEC	64 (1)	
	Sarcomatoid carcinoma	54 (0.8)	
	others	282 (4.2)	
EGFR	negative	4,208 (63.8)	2,689 (54)
	positive	2,387 (36.2)	2,295 (46)
KRAS	negative	4,779 (92.3)	3,529 (90.8)
	positive	397 (7.7)	358 (9.2)
ALK	negative	4,589 (94.2)	3,497 (92.8)
	positive	281 (5.8)	270 (7.2)

SD: standard deviation
ADC: adenocarcinoma, SqCC: squamous cell carcinoma,
SCC: small cell carcinoma, LCNEC: large cell neuroendocrine carcinoma

Supplementary Table S4: Summary of histopathologic variables

**Variables**		**Total Tumors Number (percentage)**	**Adenocarcinoma Number (percentage)**
Tumor size (cm)	Mean ± SD	3 ± 1.7	2.8 ± 1.6
Differentiation	well	205 (7.3)	191 (8.9)
	moderate	1,673 (59.3)	1,336 (62.5)
	poor	945 (33.5)	612 (28.6)
Mucinous		184 (10.4)	184 (10.4)
Signet ring cell		13 (0.8)	13 (0.8)
Primary pattern	lepidic		114 (6)
	acinar		1,240 (65.5)
	papillary		244 (12.9)
	micropapillary		50 (2.6)
	cribriform		11 (0.6)
	solid		233 (12.3)
Vascular invasion		149 (7.2)	89 (5.6)
Lymphatic invasion		640 (30.8)	470 (29.7)
Perineural invasion		113 (5.4)	51 (3.2)
Pleural invasion	P0	1,711 (80)	1,331 (79.8)
	P1	186 (8.7)	169 (10.1)
	P2	161 (7.5)	121 (7.3)
	P3	80 (3.7)	46 (2.8)
Pathologic T stage	T1	1,191 (51.5)	1,013 (56.6)
	T2	921 (39.8)	671 (37.5)
	T3	164 (7.1)	84 (4.7)
	T4	37 (1.6)	21 (1.2)
Pathologic N stage	N0	1,388 (67)	1,109 (70.7)
	N1	289 (13.9)	158 (10.1)
	N2	375 (18.1)	286 (18.2)
	N3	21 (1)	16 (1)

SD: standard deviation

Supplementary Table S5: Association Between *EGFR* Mutation and Clinicopathologic Variables

		Univariate analysis			Multivariate analysis		
		**OR**	**95% CI**	***P*-value**	**OR**	**95% CI**	***P*-value**
Sex	F vs. M	4.27	3.84-4.75	<0.001	1.83	1.56-2.16	<0.001
Age	per 1 year	0.98	0.98-0.98	<0.001	0.99	0.99-1.00	0.019
	40-64 vs. ≥ 65	1.82	1.64-2.02	<0.001	1.58	1.41-1.76	<0.001
	40-64 vs. < 40	1.83	1.27-2.67	0.002	2.19	1.48-3.28	<0.001
Smoking	Never vs. ever	4.58	4.10-5.11	<0.001	2.04	1.73-2.41	<0.001
Type							
	ADC vs. non-ADC	22.3	16.9-30.3	<0.001	14.0	10.6-19.1	<0.001
KRAS	N vs. P	40.8	19.9-103	<0.001	51.0	24.8-129	<0.001
ALK	N vs. P	24.1	12.3-56.6	<0.001	55.3	28.0-130	<0.001
Differentiation							
vs. poor	Well	3.85	2.67-5.58	<0.001	2.46	1.63-3.75	<0.001
	Moderate	3.24	2.70-3.89	<0.001	2.74	2.21-3.38	<0.001
Tumor size[a]	per 1 cm	0.88	0.82-0.93	<0.001	0.91	0.85-0.97	0.003
Mucinous[a]	N vs. P	21.1	11.7-43.1	<0.001	26.8	14.7-54.9	<0.001
Signet ring cell[a]	N vs. P	15.0	2.95-274	0.009	17.2	3.25-317	0.007
Pattern[a]							
vs. solid	Lepidic	2.03	1.27-3.24	0.003	2.18	1.30-3.69	0.003
	Acinar	3.88	2.87-5.30	<0.001	3.38	2.46-4.67	<0.001
	Papillary	3.46	2.37-5.09	<0.001	3.17	2.13-4.75	<0.001
	Micropapillary	0.96	0.47-1.86	0.901	1.05	0.48-2.19	0.898
	Cribriform	0.25	0.01-1.32	0.186	0.17	0.01-0.96	0.100

a: Analysis is performed with adenocarcinoma

OR: odds ratio, CI: confidence interval of odds ratio, M: male, F: female, ADC: adenocarcinoma, N: negative, P: positive

Supplementary Table S6: Frequency, proportion and positive rate of different type of *EGFR* mutations

	Frequency	Proportion (%)	Positive rate (%)
Exon 19 deletion	1,262	48.6	19.1
L858R	921	35.4	13.3
T790M	161	6.2	2.4
G719X	81	3.1	1.2
Exon 20 insertion	54	2.1	0.8
S768I	20	0.8	0.3
Exon 19 insertion	11	0.4	0.2
L861Q	10	0.4	0.2
E709K	9	0.3	0.1
L747P	6	0.2	0.1
L833V	6	0.2	0.09
L861R	4	0.2	0.06
S720P	3	0.1	0.04
R776H	3	0.1	0.04
A722T	3	0.1	0.04
E709A	3	0.1	0.04
V774M	3	0.1	0.04
H835L	3	0.1	0.04
Other rare mutations	36	1.4	0.5

Proportion is frequency divided by total number of positive mutations (*N*=2,599). Positive rate is frequency divided by total number of tumors with or without *EGFR* mutations (*N*=6,595)

Supplementary Table S7: Association between *KRAS* mutation and clinicopathologic variables

		Univariate analysis			Multivariate analysis		
		**OR**	**95% CI**	***P*-value**	**OR**	**95% CI**	***P*-value**
Sex	M vs. F	2.04	1.62-2.58	<0.001	1.67	1.18-2.35	0.003
Age	per 1 year	1.03	1.02-1.04	<0.001	1.03	1.02-1.04	<0.001
40-64 vs.	≥ 65	1.57	1.27-1.95	<0.001	1.72	1.38-2.14	<0.001
	< 40	0.58	0.18-1.41	0.294	0.66	0.02-1.62	0.426
Smoking	Ever vs. Never	1.92	1.55-2.39	<0.001	1.78	1.30-2.47	<0.001
Type	ADC vs.non-ADC	4.27	2.89-6.61	<0.001	7.28	4.85-11.5	<0.001
EGFR	P vs. N	0.02	0.01-0.05	<0.001	0.02	0.01-0.04	<0.001
Tumor size[a]	per 1 cm	1.21	1.11-1.33	<0.001	1.17	1.07-1.28	<0.001
Differentiation							
	Poor vs. well	2.39	1.04-6.92	0.065	1.94	0.84-5.66	0.164
	Poor vs. moderate	2.12	1.50-3.01	<0.001	1.88	1.30-2.73	0.001
Mucinous[a]	P vs. N	7.96	5.32-11. 9	<0.001	9.09	5.96-13. 9	<0.001
Pattern[a]							
vs. acinar	Lepidic	1.86	0.79-3.86	0.118	2.28	0.96-4.85	0.044
	Papillary	1.76	1.00-3.00	0.043	1.66	0.92-2.86	0.078
	Micropapillary	2.34	1.04-4.73	0.026	2.43	1.06-5.03	0.024
	Cribriform	3.64	0.19-23.1	0.243	4.04	0.20-29.0	0.226
	Solid	2.86	1.70-4.70	<0.001	2.57	1.50-4.30	<0.001

a: Analysis is performed with adenocarcinoma

OR: odds ratio, CI: confidence interval of odds ratio, M: male, F: female, ADC: adenocarcinoma, N: negative, P: positive

Supplementary Table S8: Association between *ALK* rearrangement and clinicopathologic variables

		Univariate analysis			Multivariate analysis		
		OR	95% CI	*P*-value	OR	95% CI	*P*-value
Sex	F vs. M	2.25	1.77-2.89	<0.001	1.06	0.74-1.53	0.753
Age	per 1 year	0.94	0.93-0.95	<0.001	0.95	0.94-0.96	<0.001
vs. 40-64	>= 65	0.37	0.28-0.49	<0.001	0.45	0.34-0.59	<0.001
	< 40	2.69	1.55-4.44	<0.001	2.81	1.60-4.72	<0.001
Smoking	Never vs. Ever	2.56	1.98-3.33	<0.001	1.73	1.17-2.53	0.005
Type	ADC vs. non-ADC	11.7	5.68-29.7	<0.001	6.99	3.35-17.9	<0.001
EGFR	P vs. N	0.04	0.02-0.08	<0.001	0.02	0.01-0.04	<0.001
Differentiation							
	Poor vs. well	3.31	1.33-11.0	0.023	5.61	2.20-19.1	0.001
	Poor vs. moderate	2.02	1.36-2.99	<0.001	2.54	1.67-3.86	<0.001
Pattern[a]							
vs. acinar	Lepidic	0.56	0.03-2.71	0.576	0.53	0.03-2.56	0.533
	Papillary	1.44	0.61-3.05	0.371	1.40	0.59-3.01	0.409
	Micropapillary	1.07	0.06-5.27	0.950	1.00	0.06-5.07	0.997
	Cribriform	23.9	5.67-94.8	<0.001	22.9	5.15-97.1	<0.001
	Solid	2.73	1.40-5.11	0.002	2.96	1.48-5.74	0.002
Signet ring cell	P vs. N	22.7	6.91-74.6	<0.001	20.3	5.98-5.98	<0.001
Vascular invasion	P vs. N	2.65	1.25-5.12	0.006	3.85	1.76-7.71	<0.001
Lymphatic invasion	P vs. N	2.03	1.22-3.34	0.006	2.13	1.27-3.55	0.004
Perineural invasion	P vs. N	1.83	0.69-4.02	0.170	2.96	1.08-6.85	0.019
N stage							
vs. 0	1	1.05	0.42-2.26	0.911	1.41	0.56-3.10	0.429
	2	3.84	2.28-6.45	<0.001	3.95	2.30-6.73	<0.001
	3	5.04	0.78-18.7	0.036	4.76	0.71-18.78	0.049

a: Analysis is performed on tumors with adenocarcinoma histology only
OR: odds ratio, CI: confidence interval of odds ratio, M: male, F: female, ADC: adenocarcinoma, SqCC: squamous cell carcinoma, SCC: small cell carcinoma, LCNEC: large cell neuroendocrine carcinoma, N: negative, P: positive

Supplementary Table S9: Patients having two or more primary lung tumors

	Age	Sex	Smoking	Site	Histologic type	T stage	N stage	*EGFR*		*KRAS*	*ALK*
DP01	68	M	Never	RUL	Adenocarcinoma	1a	0	P	p.E746_A750del	N	N
DP01	71	M	Never	LLL	Adenocarcinoma	1a	2	P	p.L861Q	N	N
DP02	79	F	Never	RUL	Adenocarcinoma	2a	0	P	p.L858R	N	N
DP02	79	F	Never	RLL	Adenocarcinoma	1a	0	N		ND	N
DP03	46	M	Never	RLL	Adenocarcinoma	1a	0	P	p.R776H	N	N
DP03	49	M	Never	LLL	Adenocarcinoma	1a	0	P	p.L858R	N	N
DP03	49	M	Never	RLL	Adenocarcinoma	1	0	P	p.G719C	N	N
DP04	60	M	Former	LUL	Adenocarcinoma	1a	0	P	p.E746_A750del	N	N
DP04	62	M	Former	RUL	Adenocarcinoma	1a	0	P	p.L747_P753delinsS	N	N
DP05	70	F	Never	LLL	Adenocarcinoma	1b	0	P	p.H773_V774delinsLM	N	N
DP05	70	F	Never	RML	Adenocarcinoma	1a	0	N		N	N
DP06	61	M	Former	RUL	Adenocarcinoma	1a	0	N		N	N
DP06	61	M	Former	LUL	Adenocarcinoma	1a	0	N		P	N
DP07	77	M	Former	LUL	Adenocarcinoma	1a	0	N		N	N
DP07	77	M	Former	RLL	Squamous cell carcinoma	2b	0	N		N	N
DP08	72	F	Never	LLL	Adenocarcinoma	1a	0	P	p.E746_A750del	N	N
DP08	72	F	Never	RML	Adenocarcinoma	1a	0	P	p.N771_H773dup	N	N
DP09	61	F	Never	LLL	Adenocarcinoma	2a	2	N		N	P
DP09	61	F	Never	RLL	Adenocarcinoma	1a	0	P	p.L858R	N	N
DP10	70	M	Former	RLL	Adenocarcinoma	2	0	N		N	N
DP10	72	M	Former	LLL	Adenocarcinoma	1a	0	N		N	N
DP11	74	F	Never	RUL	Adenocarcinoma	2a	0	N		ND	N
DP11	74	F	Never	LLL	Adenocarcinoma	2a	0	P	exon 19 deletion	ND	N

M: male, F: female, RUL: right upper lobe, RML: right middle lobe, RLL: right lower lobe, LUL: left upper lobe, LLL: left lower lobe, P: positive, N: negative, ND: test not done
